# Hepatitis E Virus in the Iberian Peninsula: A Systematic Review

**DOI:** 10.1007/s12560-023-09560-5

**Published:** 2023-07-11

**Authors:** Sérgio Santos-Silva, Danny Franciele da Silva Dias Moraes, Pedro López-López, António Rivero-Juarez, João R. Mesquita, Maria São José Nascimento

**Affiliations:** 1 ICBAS – Instituto de Ciências Biomédicas Abel Salazar, Universidade do Porto, Porto, Portugal; 2 State Department for the Environment of Mato Grosso (SEMA), Cuiabá, 78049-913 Brazil; 3 Grupo de Virología Clínica y Zoonosis, Unidad de Enfermedades Infecciosas, Instituto Maimónides de Investigación Biomédica de Córdoba, Hospital Reina Sofía, Universidad de Córdoba, Córdoba, Spain; 4 CIBERINFEC, ISCIII - CIBER de Enfermedades Infecciosas, Instituto de Salud Carlos III, Madrid, Spain; 5 Epidemiology Research Unit (EPIUnit), Instituto de Saúde Pública da Universidade do Porto, Porto, Portugal; 6 Laboratório para a Investigação Integrativa e Translacional em Saúde Populacional (ITR), Porto, Portugal; 7 Faculdade de Farmácia, Universidade do Porto (FFUP), Porto, Portugal

**Keywords:** Hepatitis E virus, Iberian Peninsula, Zoonotic, Infection, Systematic review

## Abstract

One of the most frequent causes of acute viral hepatitis is hepatitis E virus (HEV) causing 20 million infections worldwide each year and 44,000 deaths. Studies on HEV in the Iberian Peninsula have been increasing through time with HEV infection being identified in humans and animals. The aim of the present systematic review was to compile and evaluate all the published data on HEV from studies performed in humans, animals and environmental samples in the Iberian Peninsula. The electronic databases Mendeley, PubMed, Scopus, and Web of Science were thoroughly searched, and research published up until February 01, 2023 were included. Resulting in a total of 151 eligible papers by full reading and application of PRISMA exclusion/inclusion criteria. Overall, the present review shows that several HEV genotypes, namely HEV-1, 3, 4, and 6 as well as *Rocahepevirus*, are circulating in humans, animals, and in the environment in the Iberian Peninsula. HEV-3 was the most common genotype circulating in humans in Portugal and Spain, as expected for developed countries, with HEV-1 only being detected in travelers and emigrants from HEV endemic regions. Spain is the biggest pork producer in Europe and given the high circulation of HEV in pigs, with HEV-3 being primarily associated to zoonotic transmission through consumption of swine meat and meat products, in our opinion, the introduction of an HEV surveillance system in swine and inclusion of HEV in diagnostic routines for acute and chronic human hepatitis would be important. Additionally, we propose that establishing a monitoring mechanism for HEV is crucial in order to gain a comprehensive understanding of the prevalence of this illness and the various strains present in the Iberian Peninsula, as well as their potential impact on public health.

**Supplementary Information:**

The online version contains supplementary material available at 10.1007/s12560-023-09560-5.

## Introduction

Hepatitis E virus (HEV) infection is one of the most common causes of acute viral hepatitis, with an estimated 20 million HEV infections each year globally leading to 44,000 deaths (Nimgaonkar et al., 2018; Velavan et al., 2021; WHO 2022). HEV is classified in the family *Hepeviridae*, which has two subfamilies, *Parahepevirinae* and *Orthohepevirinae* according to the 2022 release of the International Committee on the Taxonomy of Viruses (ICTV) (Purdy et al., 2022). Members of the subfamily *Parahepevirinae* infect trout and salmon, while members of the *Orthohepevirinae* infect mammals and birds and are further classified into four genera: *Paslahepevirus*, *Avihepevirus*, *Rocahepevirus*, and *Chirohepevirus*. The genus *Paslahepevirus* includes two species, *P. balayani* that infect humans and some mammalian species (Nishizawa et al., 2021), and *P. alci*, that infects moose (Smith et al., 2020). Four major HEV genotypes (HEV-1 to HEV-4) within the species *Paslahepevirus balayani* infect humans. While HEV-1 and HEV-2 exclusively infect humans HEV-3 and HEV-4 infect humans and several other animals, mainly pigs (Smith et al., 2020). Unlike other human hepatotropic viruses, HEV has more than a dozen animal reservoirs, and HEV from pigs, rabbits, deer, camels, and rats can cross species barriers and cause infection in humans (Primadharsini et al., 2021; Wang & Meng, 2021).

HEV1 and HEV-2, predominate in developing countries are primarily spread by the fecal–oral route by drinking contaminated water while zoonotic HEV-3 and HEV-4 are worldwide and are mainly transmitted to humans through consumption of raw or undercooked pork meat or pork products or through handling infected pigs (Sooryanarain & Meng, 2019). The most common and widespread HEV genotype in Europe is HEV-3, while HEV-4 is prevalent in Japan (Pallerla et al., 2020; Takahashi et al., 2020). Most human cases of HEV-3 are zoonotic and arise from infected animals such as pigs, wild boar, deer and rabbits (Pallerla et al., 2020). Numerous studies reporting the zoonotic transmission of HEV-3 and HEV-4 have been published, especially in high-income countries where the fecal–oral route of HEV transmission is still unknown and other potential routes are being researched (Huang et al., 2016; King et al., 2018).

HEV-3 infection is frequently asymptomatic or self-limited in immunocompetent people, and often goes undiagnosed. However, HEV-3 infections in immunosuppressed patients may progress to a chronic status resulting in cirrhosis, requiring antiviral therapy (Kamar et al., 2014; Sastre et al., 2020). Several extra hepatic manifestations have been associated to HEV-3 infection, such as pancreatitis, glomerulonephritis, and neurological complications as Guillain-Barré syndrome and neuralgic amyotrophy (Kamar et al., 2017).

The increasing prevalence of autochthonous cases of hepatitis E in European countries has drawn the attention of researchers, who have been dedicated to investigating it intensively in recent years. In particular, Spain and Portugal, where dietary habits include a high consumption of pork and its derivatives, along with significant pig production, make the circulation of the HEV virus a worrying expectation.

Due to the close geographical proximity of Portugal and Spain, similar epidemiological effects that affect both nations have been found. Several studies on HEV have been performed in humans, animals and environmental samples in the Iberian Peninsula (Bes et al., 2022; Mendoza-Lopez et al., 2020; João R. Mesquita et al., 2016a, 2016b, 2016c; M. S. Nascimento et al., 2020; Rivadulla et al., 2019; Salvador et al., 2020; Santos-Silva et al., 2023), but data has not yet been the subject of any review papers. As such, and from a One Health perspective, the current systematic review aimed to compile and evaluate all the HEV published data from studies performed in humans, animals and environment in the Iberian Peninsula until February 2023.

## Material and Methods

Exhaustive searches were carried out in the electronic databases, Mendeley, PubMed, Scopus and Web of Science, including studies published until February 01, 2023. The Preferred Reporting Items for Systematic Reviews and Meta-Analysis (PRISMA) criteria were followed for the systematic review (Shamseer et al., 2015), and the studies included should necessarily be published, indexed and peer reviewed. Language barriers were not included.

The literary search used the following keywords (HEV OR Hepatitis E Virus) AND (Portugal OR Spain OR Iberian Peninsula). After reading the title and the abstract, papers that did not address the detection of HEV in Iberian Peninsula in the scope or part of the scope as well as reviews and experimental studies were excluded from this systematic review. Only articles that contained the target topic were included after being read in full due to unclear information in the title and abstract.

Two independent investigators (DFSDM and SS-S) screened the databases, and relevant information was extracted. Differences in opinions about whether to include an article were solved by consensus between the authors.

**Fig. 1:**
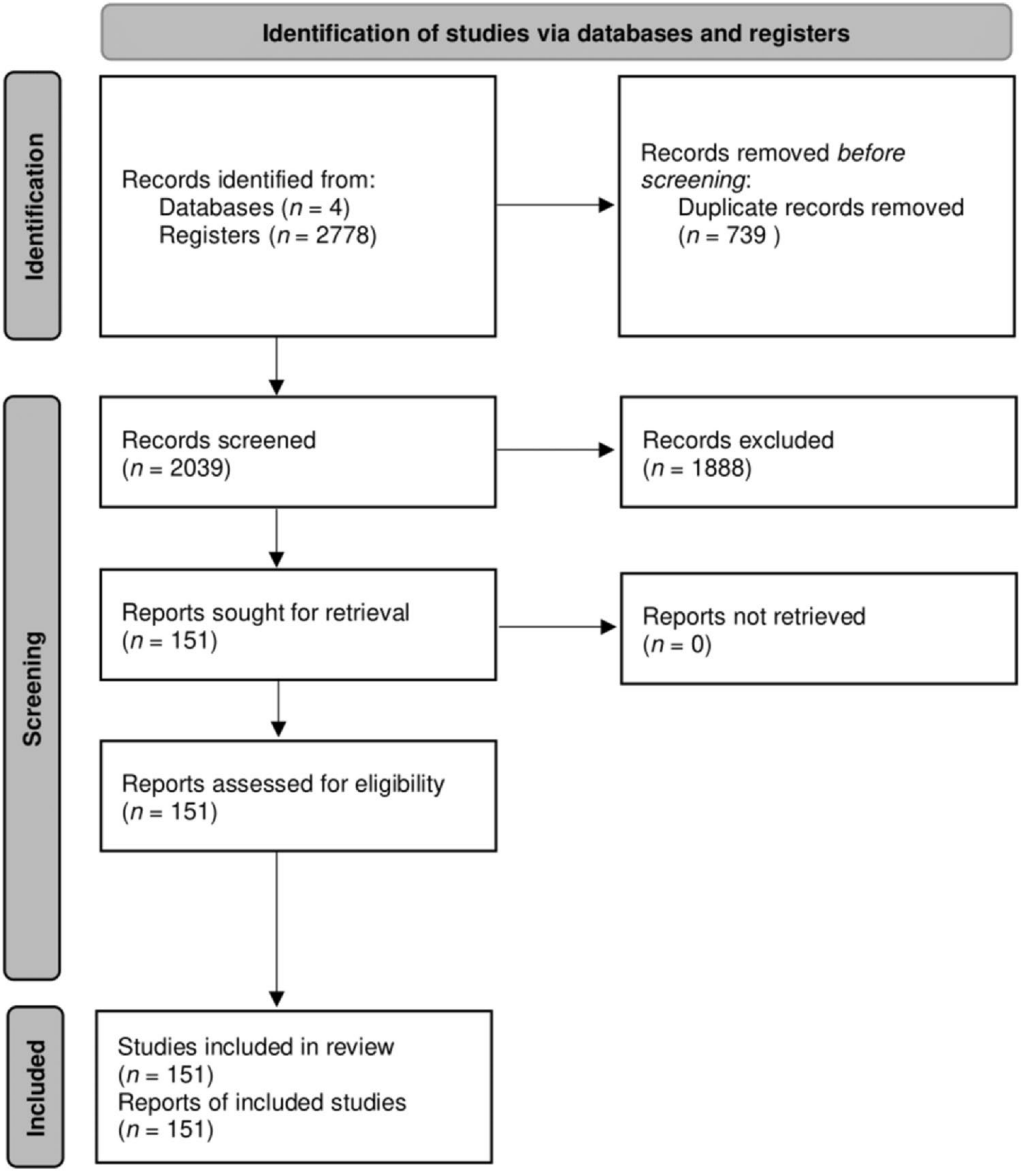
The PRISMA flow diagram presents a visual representation of the various stages in-volved in the record selection process, outlining the strategies used for inclusion and exclusion

Once the databases were purged of duplicate papers (n = 739), a thorough analysis of exclusion criteria was conducted, which resulted in the identification 1888 unrelated re-search studies. By applying both inclusion and exclusion criteria, it was possible to narrow down the potential papers suitable for the systematic review to 151 (Fig. 1).

## Results

A total of 2778 papers were retrieved from the four electronic databases used for the search. After assessing, by full reading, all 151 papers were considered eligible and were included. A summary of all the HEV studies in humans, animals (including products of animal origin for human consumption) and environment samples, as well as the key points of each of these papers are described in Supplementary Tables 1–5.

### HEV in Humans

#### Molecular Studies in Human Samples

A total of 59 molecular studies of HEV in human using a molecular approach were performed in the Iberian Peninsula, namely 11 in Portugal and 48 in Spain (Supplementary Table 1).

In Portugal, the first study reporting an autochthonous human infection with HEV-3 (subgenotype 3a) was published in 2013 in a man with a severe acute hepatitis complicated by Guillain-Barré syndrome being also the first case in Portugal describing the association of hepatitis E with a neurological disorder (Santos et al., 2013). HEV-3 was also the cause of severe acute hepatitis, that required hospitalization, in two women with an autoimmunity background (Nascimento et al., 2020; Vieira et al., 2013). HEV-3 (subgenotype 3c) was identified in liver and renal transplant recipients with chronic hepatitis, marking the first report of chronic hepatitis E in Portugal (Carmo et al., 2016). HEV was also found in kidney transplant patients with acute hepatitis (Casas et al., 2011a). Additionally, ribavirin has been evaluated for its effectiveness in treating this patient population (Carmo et al., 2016). The persistence of HEV infection was also reported in a patient with myelofibrosis (Filipe et al., 2022; Ribeiro da Cunha & Marques, 2021) but no chronic infections was observed in a Portuguese cohort of HIV (Filipe et al., 2022; Ribeiro da Cunha & Marques, 2021).

HEV-1 was identified in emigrants from India that were hospitalized with a severe acute hepatitis being the first imported cases of hepatitis E caused by genotype 1 published in Portugal (João R. Mesquita et al., 2018). In a nationwide study aiming to compare the incidence of HEV infection among blood donors in Portugal and Germany, a prevalence of 0.02% was found in Portuguese donors, an incidence five times smaller than in Germany (Healy et al., 2022).

In Spain, studies on HEV have started earlier then Portugal. The first study on HEV was published in 2000 and focused on patients with acute hepatitis. The virus was detected in human sera with a nucleotide similarity of 92–94% with the HEV RNA being found in slaughterhouse sewage (Pina et al., 2000). Another study on patients with acute hepatitis found two HEV strains that were classified as genotype 6 (M. T. Pérez-Gracia et al., 2004). Seven years later a study referred for the first time HEV-3, subgenotype 3f in a sample from a slaughterhouse worker with acute hepatitis (Maria Teresa Pérez-Gracia et al., 2007). A study performed in Barcelona identified HEV-1 as the cause of the acute hepatitis in a traveler from an endemic area (Ethiopia) and HEV-3 as the agent of the two cases of acute autochthonous hepatitis E (Maria Buti et al., 2004) and in the same region another study detected HEV-3 also in an acute hepatitis patients (Jung et al., 2018). Furthermore, a study reported the presence of HEV RNA in the absence of clinical symptoms related to viral acute hepatitis (ALT and AST also normal) (Mateos-Lindemann et al., 2012). A study on patients with acute hepatitis found viral etiology evidence in 13.9% (13/95) of the cases with HEV-1 and HEV-4 being identified in travelers and HEV-3, subgenotype 3f detected in autochthonous cases (Fogeda, Ory, et al. 2009). Another nationwide study, performed with samples collected between 2009 and 2019 from patients with acute hepatitis A–C, found 409 samples out of 5197 positive for HEV-3, subgenotypes 3c, 3f and 3 m (Muñoz-Chimeno et al., 2022). For the first time, a woman in Madrid who was not pregnant and was using hormonal contraceptives that mimic pregnancy status, experienced severe hepatitis E caused by the HEV-3 subgenotype 3f (Lindemann et al., 2010). A study performed in HIV patients from Madrid and Seville with severe immunosuppression was the first to show that ribavirin treatment may be transiently efficacious against the rapid progression to chronic hepatitis E in these patients (Neukam et al., 2013). Another study also in HIV patients concluded that mutations in the progesterone receptor may reduce the symptoms of acute hepatitis E and protect against infection having found the presence of HEV RNA and/or IgM in 66.7% (26/39) of homozygous wild type patients, 20.5% (8/39) in heterozygous and 12.8% (5/39) in homozygous (López-López et al., 2019). Additionally, in Catalonia, two different cohorts were evaluated: one including patients with self-limited acute hepatitis E comparing with blood donors positive for HEV RNA, in which it was demonstrated that IgM antibodies can be present after 34 months even when HEV RNA was not detectable much earlier (Riveiro-Barciela et al., 2020). Furthermore, in the same region a study reported two cases of acute symptomatic HEV genotype 3 infection in patients carrying anti-HEV IgG, after successful completion of HCV therapy (Antonio Rivero-Juarez et al., 2017c). In that study HEV reactivation or reinfection was questioned.

In a study involving a patient with an immunosuppressed system, it was found that getting infected with Hepatitis E virus (HEV) through cryosupernatant plasma may trigger Thrombotic Thrombocytopenic Purpura (TTP). This patient was treated with ribavirin, HEV infection was overcome and the TTP symptoms improved (Riveiro-Barciela et al., 2018). Another study found that the use of ribavirin (12 weeks) for the treatment of HEV infection in organ transplant patients was effective, in that study HEV-3f was detected (Antonio Rivero-Juarez, Vallejo, et al. 2019).

Studies aiming the detection of HEV RNA in Spanish blood donors, found HEV-3 in this healthy population, namely subgenotype 3f in three out of 9998 (0.030%) in blood donors from Catalonia (Sauleda et al., 2015) and subgenotypes 3c and 3f in 151 out of 655,523 (0.023%) in samples collected between 2017 and 2020 (Bes et al., 2022). In another study HEV-3 was detected in four out of 11,313 (0.035%) in blood donors from Castilla la Mancha (Antonio Rivero-Juarez et al., 2019a). In 2017, in Catalonia it was published the first report in Europe of a full-blown acute hepatitis E due to HEV-3 in an immunocompetent adult associated with the transfusion of red blood cells (Riveiro-Barciela et al., 2017). In Galicia, it was also reported for the first time a case of acute meningitis secondary to autochthonous HEV-3 infection in an immunocompetent (Rodríguez-Castro et al., 2018). HEV-3 was also responsible for a familial hepatitis E outbreak linked to the consumption of hunted wild boar contaminated meat in Andalusia (Rivero-Juarez et al., 2017a). Follow up studies conducted in Cordoba with saliva and sera samples from patients with acute hepatitis and liver from organ donors in which authors suggest that acute HEV infection could be diagnosed by assessing viral load in saliva and detection of HEV genotype 3f in liver samples suggesting the potential risk of HEV transmission through liver allograft transplants (Rivero-Juarez et al., 2018a, 2018b). Another study detected HEV in patients with drug-induced liver injury (DILI) tested positive for anti-HEV IgM (Sanabria-Cabrera et al., 2021).

### Serological Studies in Human Samples

A total of 66 serological studies of HEV in human samples were performed in Iberian Peninsula, namely 17 in Portugal and 49 in Spain (Supplementary Table 2).

In Portugal, the first study was performed in 1997 in hemophiliacs and blood donors reporting a seroprevalence of anti-HEV antibodies of 3.8% (2/52) and 3.2% (11/341), respectively (Araújo et al., 1997). In the same year, another study in blood donors from the North of Portugal reported a seroprevalence of 2.5% (37/1473) (Queirós et al., 1997). The first report of an acute autochthonous hepatitis E in Portugal was published in 2012 and was only based on serological markers, namely in the presence of IgM and IgG anti-HEV antibodies and in the epidemiological data, there was no travel history (Duque et al., 2012). A study showed that there was effective transport of anti-HEV IgG through the placenta and the presence of locally occurring HEV in Portugal (J R Mesquita et al., 2013). It was also the presence of anti-HEV IgM that led to suspect an autochthonous HEV infection in an immunocompetent man who had never travelled outside of Portugal. Further hospitalized due to a severe acute hepatitis complicated by Guillain-Barré syndrome (L. Santos et al., 2013). Also two renal transplant recipients with chronic hepatitis were initially diagnosed with an hepatitis E by the presence of anti-HEV IgM (Breda et al., 2014). The screening of 71 archived sera from a Portuguese pediatric cohort (1–13 years old) collected in 1992–1995 found anti-HEV IgG in two children and anti-HEV IgM in one, suggesting that HEV was circulating in the pediatric population of Portugal in the early 1990s (Mesquita et al., 2016b). In a nationwide serosurvey in the general population of Portugal, launched in 2015–2016, an overall HEV IgG seroprevalence of 16.3% was found, seroprevalence increased with age, from 0.6% in the group of children to 30.1% in people older than 70 years (Nascimento et al., 2018). The seroprevalence showed also to vary geographically with generally higher seropositivities (25–30%) in the most rural areas of Portugal (Nascimento et al., 2018).

A study that has compared the prevalence of anti-HEV IgG in pet veterinarians and in general population found no significant difference between the two groups, 9.7% (36/373) and 13.3% (16/120), respectively, concluding that occupational exposure to pets does not increase the likelihood for HEV infection (Mesquita et al., 2014). But a study conducted in the Centre and North regions with sera collected in 2015 found a significantly higher anti-HEV IgG seroprevalence in workers occupationally exposed to swine (30.7%) compared to the general population (19.9%), showing that these workers have an increased risk of HEV infection (Teixeira et al., 2017). Also, in a study conducted in the Centre Region of Portugal with sera collected in 2017 the HEV seroprevalence in workers exposed to ovine (29.3%) was found to be significantly higher when compared with population controls (16.1%) which suggests an increased risk for HEV infection in these workers (Mesquita et al., 2020).

In Spain, the first study was conducted in Barcelona in samples collected in 1989–1991 from patients with acute hepatitis, being all negative for IgM and IgG anti-HEV antibodies (M Buti et al., 1994). A study in samples collected in 1993–1994 from children (13–15 years old) living in rural areas showed IgG ani-HEV in 5.3% (5/95), being this seroprevalence higher than other European countries (Montes Martínez & Agulla Budiño, 1996). In 1997, a seroprevalence study carried out in Madrid in blood donors and hemodialyzed patients detected IgG anti-HEV in 6.3% (4/63) and 2.8% (25/863), respectively, suggesting that the prevalence of HEV in Spain, as indicated by the presence of anti-HEV IgG antibodies in blood donors, is comparable to that in other Western European countries and that hemodialysis patients should take into account the possibility of HEV infection. (Marla Luisa Mateos et al., 1997). A study from Barcelona in samples collected in 1989–1999 from patients with acute hepatitis found IgG anti-HEV in 21.6% (8/37), the causal role of HEV in acute hepatitis cases among the Spanish population was investigated, and only non-B non-C acute hepatitis patients were tested for the presence of Anti-HEV antibodies (Pina et al., 2000). In 2000, a seroprevalence study conducted in Madrid in blood donors and immigrants from sub-Saharan Africa showed a prevalence of antibodies anti-HEV 1.9 times higher in immigrants (Tarragó et al., 2000). In Catalonia, a community-based seroepidemiological survey of HEV infection carried out in general population (15–75 years old) in samples collected in 2002 detected IgG anti-HEV in 7.3% (96/1,280), being the first large Spanish study of HEV seroprevalence (Maria Buti et al., 2006). It was also in Catalonia that was conducted the first large seroprevalence (4.6%) study in healthy children, that demonstrated exposition to HEV in early childhood (Maria Buti et al., 2008) and in pregnant women that presented a prevalence of IgG anti-HEV of 5.4% (82/1,517) (Maria Buti et al., 2010). In Barcelona, sera collected between 2004 and 2007 from patients with acute hepatitis showed 100% positivity to IgG anti-HEV (Rodriguez-Manzano et al., 2010). In Madrid was reported the first case of an autochthonous fulminant hepatic failure associated to HEV in a woman, that showed to be positive for IgM and IgG anti-HEV (Lindemann et al., 2010). Furthermore, a study detected IgM/IgG anti-HEV in patients with sporadic autochthonous hepatitis E and imported hepatitis E cases (María Luisa Mateos et al., 2006). In Valencia seroprevalence was compared between pig workers and blood donors, with IgG anti-HEV detected in 18.6% (21/113) and 4% (4/99), respectively, supporting HEV infection as an occupational disease (Galiana et al., 2010). In Madrid a study with HIV patients showed that they are at high-risk for HEV infection, identifying 6.6% positive for IgM anti-HEV (3/45) and 10.4% for IgG anti-HEV (45/448), with no difference regarding age, gender or CD4 count (Mateos-Lindemann et al., 2014). Furthermore, in Madrid a high seroprevalence was found in HIV-HCV co-infected patients. In that study, HIV infected patients had no increased risk for HEV infection (López-Fabal & Gómez-Garcés 2015). In 2013, a study was conducted comparing different assays, which showed different results. It was reported that being male was statistically significant as a risk factor compared to females (Sauleda et al., 2015). The first study conducted in Leon tested sera collected between 2008 and 2014 from patients with acute hepatitis E, confirming the presence of IgM anti-HEV in 95.65% (22/23) and IgG in 100% (23/23) (Monteserín-Ron et al., 2017). In a longitudinal study performed in Cordoba in HIV patients it was found that living in a rural habitat was a risk factor for HEV seroconversion having 0.8% (5/627) of the patients seroconverted for IgM anti-HEV and 5.7% (36/627) for IgG anti-HEV (A Rivero-Juarez et al., 2017b). One study from Málaga, reporting a retransplant due to fulminant hepatic failure due to HEV infection in a patient, found a seroconversion for both anti-HEV antibodies, IgM and IgG (Tenorio González et al., 2018). In the study of Galicia, that described an acute meningitis secondary to autochthonous HEV-3 infection in an immunocompetent patient, the serologic acute phase marker, IgM anti-HEV was also detected (Rodríguez-Castro et al., 2018). A study conducted in Cordoba showed that homozygous individuals with mutations in the progesterone receptor were associated with a lower HEV seroprevalence (López-López et al., 2019). In a longitudinal study performed in Ciudad Real in 4 blood donors with HEV infection a late seroconversion was observed, with the appearance of IgM and IgG anti-HEV only after 2–4 months in 1 out of 4 donors and 4 out of 4, respectively (Antonio Rivero-Juarez et al., 2019a). In a study done in Barcelona it was found that HEV was the second leading cause of acute hepatitis (Llaneras et al., 2021). In a long-term study conducted in Catalonia in patients with self-limited acute hepatitis E and HEV RNA-positive blood donors reported for the first time the durability of anti-HEV IgM after a self-limited acute hepatitis E (Riveiro-Barciela et al., 2020). In a long-term longitudinal study performed in Cordoba, sera were collected between 2012 and 2020 from HIV/HCV-coinfected cirrhotic patients in order to evaluate the kinetic of HEV antibodies, having been detected at the baseline 0% (0/75) IgM anti-HEV and 17.3% (13/75) IgG anti-HEV. After 5.1 years a seroconversion of 2.7% (2/75) was observed for IgM anti-HEV while for IgG anti-HEV two seroconversions and five seroreversions were registered, totalizing 13.3% (10/75) IgG anti-HEV at the end of the study (López-López et al., 2022). Furthermore, a study showed that clotting-factor concentrates are not at risk for causing HEV infection (FLORES et al., 1995).

### HEV in Animals

#### Molecular Studies in Animal Samples

A total of 42 molecular studies of HEV in animal samples were performed in Iberian Peninsula, namely six in Portugal and 36 in Spain (Supplementary Table 3).

In Portugal the first study that reported HEV RNA in animals was conducted in pigs of all the four age groups (weaners, growers, fatteners and sows) from farms of the Centre Region of the country, having found a high circulation of HEV-3, namely in fatteners (the ones entering the food chain for consumption) with 32% of positivity (A. Berto et al., 2012a). Another study carried out with HEV positive stool samples from pigs founded a virus transmission rate of 0.037 day^−1^, an average infectious period of 101 days and a reproductive number (R_0_) of 3.7 (Alessandra Berto et al., 2012b). The first report of HEV in boar meat intended for human consumption was reported in 2016, 25% of livers (20/80) and 10% of stools (4/40) were positive for HEV, that clustered with sequences classified as HEV-3, subgenotype 3e (J. R. Mesquita et al., 2016a). A very recent study carried out in stools from wild boars of Portugal found 2.8% (4/144) positive samples for HEV RNA, with the first detection of the novel HEV subgenotype 3m in wild boar population in this country (Santos-Silva et al., 2023). In other free-living wild animals, more specifically deer, HEV-3 has also been detected in stools, being typed as subgenotype 3e (Moraes et al., 2022).

The first study to search HEV in animals in Spain detected high levels of HEV seroprevalence in their herds, but no evidence of HEV was found (Pina et al., 2000). Furthermore, in sera and stool samples from pigs, 6 out of 12 stool samples were positive for HEV RNA with genotype 3 of HEV typed (Clemente-Casares et al., 2003). In a study carried out in Valencia, that evaluated the shedding of HEV in stools of pigs at different stages of production concluded that HEV is most excreted in the first month of feeding (Fernández-Barredo et al., 2006). In another study conducted in naturally infected pigs with different pathological conditions, HEV detection showed higher sensitivity in bile, lymph node and liver respectively, being characterized as HEV-3 (Deus et al., 2007). The first report of HEV-3 detection in wild boar was published in 2008 (Deus et al., 2008b). In a later study carried out in stools collected in 2010–2011 from pigs, sows and boars, HEV was found negative in 144 included samples from sows in Spain and HEV-3 was detected in only 1 out of 23 (4.3%) of boar samples (Alessandra Berto et al., 2012b). The transmission of HEV through the ingestion of pork meat was demonstrated by phylogenetic analysis which revealed the same genotype 3, subgenotype 3f, between the patient´s plasma and the piece of pork meat (Riveiro-Barciela et al., 2015). Another study linked the consumption of wild boar meat to a familial hepatitis E outbreak, typing HEV as genotype 3 (A. Rivero-Juarez et al., 2017a). Another study in wild boars detected HEV in 6.8% (4/58) of liver samples and in 1.7% (1/58) of sera samples, but no typing was performed (María A. Risalde et al., 2017). A big study of HEV detection in sera samples from pigs (fatteners and sows) showed an overall prevalence of 16.5% (172/1040) highlighting higher global prevalence in extensive (23.9%) than in intensive (12.6%) farming (Lopez-Lopez et al., 2018). Another study showed a high prevalence of HEV RNA in wild boars sera, discussing the possibility of a seasonal pattern in the occurrence of HEV in wild environments (Antonio Rivero-Juarez et al., 2018c). It was also in sera samples from wild boars, namely in the only one out of 99 (1%) positive, that the subgenotype 3r of HEV was identified, being considered an emerging zoonotic subtype in Europe (Caballero-Gómez et al., 2019a). The most recent study conducted in naturally infected wild boar detected HEV RNA in different biological samples, namely in sera (4 positives out of 191), in liver (3 positives out of 4), in hepatic lymph nodes (3 positives out of 4) and in testis (1 positive out of 4), having been typing as HEV-3, subgenotype 3f (María A. Risalde et al., 2022). A study conducted in Catalonia reported for the first time avian HEV in 0.76% (2/262) of sera samples from flocks and in 1.6% (5/300) of sera bank samples (Peralta et al., 2009a). Other study detected the presence of HEV in sausages, typing as HEV-3 subgenotype 3f indicating a potential risk of transmission of this virus by consumption of pork product (Di Bartolo et al., 2012).

The first HEV study in rodents was performed in Andalusia using liver samples from black rats collected in 2015–2016, having found 4% (2/50) samples positive for *Orthohepevirus C* genotype 1 (Ryll et al., 2017). The first study in equids was carried out in sera from horses, donkeys and mules, collected between 2010 and 2014, with HEV-3 being detected in 0.4% (3/692) of horses, 1.2% (1/86) of donkeys and 3.6% (3/83) of mules, further typing as subgenotypes 3e, 3f, 3 g (García-Bocanegra et al., 2019). The first study in wild rabbits and Iberian hares was focused in liver samples but HEV was not detected (Caballero-Gómez et al., 2020). A study that correlated HEV occurrence with some enteroparasites suggested that extracellular *Giardia duodenalis* and *Blastocystis* sp. might have a protective effect on HEV acquisition in swine (Antonio Rivero-Juarez et al., 2020), with a follow up study performed by the same group showing that the existence of external enteroparasites is associated with a protective effect on the likelihood of obtaining HEV, while internal enteroparasites appear to have the opposing effect by promoting HEV contamination (Rivero-Juárez et al., 2022). Another study evidenced that liver transudate could be used for HEV RNA detection (Navarro et al., 2020). A study reported HEV RNA in 27.9% (11/29) of *Hyalomma lusitanicum* ticks feeding on wild boars and in 34.5% (10/29) of wild boar serum samples, further typing as HEV-3 subgenotype 3f (Antonio Rivero-Juarez et al., 2021).

The molecular survey performed in sera from dogs and cats showed no presence of HEV RNA in these species (Caballero-Gómez et al., 2022b). The only study that reported HEV in *Iberian lynx,* used serum, liver and stool samples but HEV was only detected in stool samples (1 out of 73) being further typed as HEV-3, subgenotype 3f (Rivero-Juarez et al., 2022). HEV was not found in sera samples from sheep and goats of Andalusia (Caballero-Gómez et al. 2019a).

#### Serological Studies in Animal Samples

A total of 29 serological studies of HEV in animal were performed in the Iberian Peninsula, namely three in Portugal and 26 in Spain (Supplementary Table 4).

In Portugal, the first study searching for antibodies anti-HEV in animals was carried out in 2018 in wild boar samples of the Alentejo Region, having anti-HEV (IgM/IgG/IgA) been detected in 14% (4/29) of animals (Gonçalves et al., 2018). In wild rabbits from the same region, anti-HEV (IgM/IgG) antibodies were found in 4.1% (3/74) (Lopes & Abrantes, 2020) and in sheep of the Central Region of Portugal IgG anti-HEV was detected in 16.6% (15/90) (Mesquita et al., 2020).

In Spain, the first serological study in animals was performed in 2000 and in sows, boars and piglets of Catalonia, having found IgG anti-HEV in 23.1% (6/26) of sows, in 29.1% (7/24) of boars and in 20% (2/10) of piglets (Pina et al., 2000). Another study detected IgG anti-HEV in 13.7% (10/73) of pigs (Clemente-Casares et al., 2003). A study in pigs with postweaning multisystemic wasting syndrome (PMWS) with hepatitis (a) and without hepatitis (b), have compared the IgG anti-HEV seroprevalences with pigs without PMWS with hepatitis (c) and without hepatitis (d) having found seroprevalences of 57.8% (37/64) in (a), 40.0% (20/50) in (b), 23.1% (3/13) in (c) and 30.3% (10/33) in (d), concluding that HEV can be a primary agent of subclinical hepatitis (Martín et al., 2007). In a seroprevalence study in pigs on commercial farms in Spain, IgM anti-HEV was detected in 28.2% (118/418) of animals and IgG anti-HEV in 41.9% (184/439) (Seminati et al., 2008). The first report of HEV infection in wild boar in Spain found a seroprevalence (IgM/IgG/IgA) of 42.7% (64/150) (Deus et al., 2008b). Furthermore, in the Northeast Region it was shown IgG/IgM/IgA anti-HEV in sows and only IgG in piglets (Deus et al., 2008a). In a retrospective serological study carried out in sera samples from pigs collected between 1985 and 1997 a high IgG anti-HEV seroprevalence of 48.4% (1390/2871) was found (Casas et al., 2009). In a study that aimed to compare muscle fluid with serum for the detection of antibodies in slaughter pigs, anti-HEV IgG was detected in 64% (43/67) in both biological fluids, which suggested that muscle fluid could be used as an alternative to serum (Casas et al., 2011b). A longitudinal study of HEV infection in farrow-to-finish swine herds of Catalonia showed IgM anti-HEV in 15% (18/119) of pigs and IgG anti-HEV in 59% (70/119) (Casas et al., 2011a). Another study in adult and young pigs showed an overall seroprevalence of 20.4% IgG anti-HEV (233/1141) (Jiménez De Oya et al., 2011). One nationwide animal serological study of HEV on wild boar was performed in 2012, and detected IgG anti-HEV in 26.3% (248/942) of animals, having seroprevalences showed to be higher in fenced animals than those of open sites (Boadella et al., 2012). The first study in spray-dried porcine plasma showed the presence of anti-HEV antibodies in all the samples tested, 85 positives out of 85 (100%) (Pujols et al., 2014). Another study analyzed samples from pigs, wild boars and red deers and detected anti-HEV in 43.7% (21/48), 57.4% (62/108) and 12.8% (9/70), respectively (Kukielka et al., 2016). Further studies on wild boar, namely in Andalusia carried out in juvenile, sub-adults and adult animals detected an overall anti-HEV seroprevalence of 5.2% (3/58) (María A. Risalde et al., 2017). Another study on wild boars from the same region detected anti-HEV in 57.6% (57/99) of animals, having adults and subadults presented higher seropositivities than yearling animals (Caballero-Gómez et al., 2019a). Still in wild boar, but from a different region, IgM/IgG anti-HEV antibodies were detected in 59% (112/190) of animals (Wang et al., 2019). The second and last nationwide study in pig sera samples detected IgG anti-HEV in 73.3% (33/45) of animals (García et al., 2020). In a study that aimed to compare liver transudate with serum for the detection of antibodies in pigs and wild boars, anti-HEV was detected in 68% (85/125) of sera samples and in 61.6% (77/125) in liver transudate samples, concluding that liver transudate could be used as an alternative matrix to serum for the detection of anti-HEV antibodies (Navarro et al., 2020). A longitudinal long-term survey of HEV exposure in wild boar reported IgG anti-HEV in 46.7% (327/700) of animals (Barroso et al., 2021).

A study in domestic animal species and rodents was performed in Catalonia and included cows, sheep, goats, and others animal species as cats, wild rodents and pigs, and have used a genotype 3-based ELISA for the detection of anti-HEV antibodies, having IgG anti-HEV been detected in all groups except cows and wild rodents (Peralta et al., 2009b). Other study was conducted in little ruminants in Andalusia, finding anti-HEV in 2.1% (5/240) of sheep and in 13.8% (33/240) of goats, showing that these animals are naturally but not equally exposed to HEV (Caballero-Gómez et al., 2022a). Other study in chickens was from Catalonia and reported anti-HEV IgG antibodies in 89.7% (26/29) of samples, revealing a widespread infection of avian HEV (Peralta et al., 2009a). Furthermore, a serological study in Iberian red deer carried out in Spain detected IgG anti-HEV in 10.4% (101/968) of animals (Mariana Boadella et al., 2010). A serosurvey of HEV infection was carried out in non-human primates from zoos of Spain, revealing a prevalence of IgG ani-HEV of 4.4% (8/181) (Caballero-Gómez et al., 2019b). Moreover, a serological survey in dogs and cats in Spain was conducted in Cordoba, having anti-HEV antibodies been detected in 9.9% (15/152) of dogs and in 2.8% (4/144) of cats (Caballero-Gómez et al., 2022b). The only serological study in Iberian lynx performed in animals from the Central, South and Southwest regions of Spain found a prevalence of anti-HEV of 18.2% (50/275) (Caballero-Gómez et al., 2022c).

### HEV Studies in Environmental Samples

A total of 23 studies of HEV in environmental samples were performed in Iberian Peninsula, namely three in Portugal and 20 in Spain (Supplementary Table 5).

In Portugal, the first study is from 2018, and was performed in influent and effluent wastewater samples from all over the country having 3.3% (2/60) been positive for HEV-3, subgenotype 3i and 3f (Matos et al., 2018). In another study conducted in the Centre of Portugal in surface and drinking water found HEV-3 in 77.8% (21/27) and 66.7% (24/36) samples, respectively (Salvador et al., 2020). HEV-3 was also detected in 2 out of 4 sea urchins collected in the North of Portugal (Santos-Ferreira et al., 2020).

In Spain the first study is from 1998, and was performed in raw sewage from Barcelona having HEV been detected in 2.7% (1 /37) of samples (Pina et al., 1998). A study performed in sewage from porcine slaughterhouses found HEV in one sample out of 12 (8.3%), that showed to be similar to HEV identified in serum from humans with acute hepatitis (Pina et al., 2000). Several studies were carried out in urban sewage water from Barcelona having HEV-3 been detected in 43.5% (20/46) (Clemente-Casares et al., 2003), 100% (4/4) (Albinana-Gimenez et al., 2006) and 17.6% (6/34) of samples (Clemente-Casares et al., 2009). In sewage from Valencia and Barcelona detected a total prevalence of 32% (29 /91) (Rodriguez-Manzano et al., 2010). Also in Barcelona HEV-3 was detected in 25% (1/4) of slaughterhouse biosolids but it was not found in river water and sludge samples (Albinana-Gimenez et al., 2006). Another study found 50% (2/4) in biosolid, and 40% (2/5) in slaughterhouse (Clemente-Casares et al., 2009). Besides HEV-3, HEV-1 similar to strains circulating in India has been detected sporadically in sewage from Barcelona (Clemente-Casares et al., 2009) and Valencia (Rodriguez-Manzano et al., 2010). A study carried out in influent raw sewage and secondary treatment effluent found HEV at low and similar levels in both influent and effluent wastewaters, namely 13.5% (5/37) and 12.5% (4/32), respectively (Rusiñol et al., 2015). The presence of HEV was also reported in 71.4% (10/14) of sewage samples from Valencia collected in 2017, that further typing identified them to be of HEV subgenotype 3f. In the same study no HEV was found in irrigation water and lettuce samples (Randazzo et al., 2018). Another study detected HEV in the influent wastewaters of the four wastewater treatment plants from Valencia collected in 2018–2019 (Cuevas-Ferrando et al., 2020). Two more studies have been conducted in environmental samples, one in drinking water, reservoir water, groundwater, river water, raw sewage, conventional activated sludge and reclaimed (wetland), with HEV being detected only in 1 out of 12 (8%) groundwater samples (a porcine contamination was identified) and in 1 out of 12 (8%) raw sewage (Rusiñol et al., 2020a) while the other study was performed in distribution water, reservoir water, groundwater, river water, wetland, raw sewage having HEV been detected in 1 out of 12 (8%) groundwater and 1 positive out of 12 (8%) raw sewage samples, this time, using metagenomic analysis (Rusiñol et al., 2020b). Two studies found HEV in other environmental samples, namely in 50% (8/16) of raw manure and in 6.2% (1/16) of animal drinking water (Fernández-Barredo et al., 2006) and in 59% (10/17) of manure-ditch from pigs samples (Fernández-Barredo et al., 2006). In a study that was searching for other human enteric viruses in coquina clams imported fom Peru that were associated with a large hepatitis A outbreak, HEV was not detected (Bosch et al., 2001). In 2012 a study in Galicia detected HEV in mussel tissue in 6% (3/51) of samples, being the first report of HEV contamination of commercial mussels at retail level in Spain (Diez-Valcarce et al., 2012). In another study with bivalves of Galicia, HEV was detected in 14.8% (12 /81) of mussels (Mesquita et al., 2016c) and in 24.4% (41/168) of bivalve molluscs (Rivadulla et al., 2019), being genotyped as HEV-3, subgenotype 3e (Rivadulla et al., 2019). In the Eastern Region of Spain, a big study failed to detect HEV in any final product (compost) destined to be commercialized as a soil fertilizer, which shows that composting is a suitable method to eliminate HEV and to reduce the transmission of HEV from pigs to humans (García et al., 2014). The first study reporting the presence of anti-HEV antibodies and HEV RNA in spray-dried porcine plasma (SDPP) from Spain identified 22.4% (11/49) contaminated samples (Pujols et al., 2014). The evaluation of the microbiological quality of reclaimed water produced from a lagooning system (lagooning influent, lagooning effluent and wastewater) did not detected HEV (Fernandez-Cassi et al., 2016).

## Discussion

In the Iberian Peninsula, human HEV studies first began in the mid-90 s. Starting in the year 2000, specifically with the discovery of cases of zoonotic and autochthonous transmission in industrialized countries, there was an increase in studies on the hepatitis E virus (HEV) in Europe, which also occurred in countries such as Portugal and Spain. The majority of these initial studies were carried out in patients with acute hepatitis, where HEV infection was confirmed based on the serological diagnosis (Buti et al., 1994; Maria Buti et al., 1995; Duque et al., 2012). The first papers were also focused on the detection of anti-HEV antibodies in blood donors in an attempt to evaluate the HEV seroprevalence of the population, with studies starting in 1997 in Portugal and even earlier in Spain, in 1994 (Araújo et al., 1997; Macedo et al., 1998; Mateos et al., 1998; Marla Luisa Mateos et al., 1997; Queirós et al., 1997; Tarragó et al., 2000). Several HEV seroprevalence studies were carried out throughout the years in the Iberian Peninsula. Some have been performed in blood donors, having found very similar HEV seroprevalences in both countries, ranging between 3.2 and 4% in Portugal (Araújo et al., 1997; Macedo et al., 1998; Queirós et al., 1997) and 2.8–6% in Spain (Galiana et al., 2010; Mateos et al., 1999; Marla Luisa Mateos et al., 1997; Tarragó et al., 2000). Other studies have been carried out in general population, having found seroprevalences ranging from 0.4 to 19.9% in Portuguese population (Mesquita et al., 2018; João R Mesquita et al., 2020; Nascimento et al., 2018; Oliveira et al., 2017; Teixeira et al., 2017) but higher HEV seroprevalences in Spanish population, namely 1.4–37% (Abe et al., 2006; Maria Buti et al., 2006, 2008; López-Izquierdo et al., 2007; Rivera et al., 2000). Several studies in the Iberian Peninsula have shown an association between HEV infection and the occupational exposure to livestock, namely workers exposed to swine and ovine given the higher seroprevalences compared with the non-risk control groups (Galiana et al., 2010; Llovet et al., 2020; Mesquita et al., 2020; Maria Teresa Pérez-Gracia et al., 2007; Teixeira et al., 2017). Indeed, this association has also been suggested in other countries, such as the United States and Taiwan (Hsieh et al., 1999; Meng et al., 2002). Seroprevalence studies performed in general population and in rural child population show higher HEV seroprevalences associated to the place of residence (Montes Martínez & Agulla Budiño, 1996; Nascimento et al., 2018), while others did not find this association (Maria Buti et al., 2008, 2010). Differences in exposure of human populations to animal reservoirs and zoonotic risk could be the cause of this data discrepancy. However, the differences of HEV seroprevalences reported in the different studies of Iberian Peninsula have to be interpreted with caution, since studies were carried out with several years apart and have used various immunoassays which vary considerably in their sensitivity and specificity that could affect the seroprevalence results (Hartl et al., 2016),

In the 2000’s several studies from Spain started to identify HEV-3 in patients with autochthonous acute hepatitis (Maria Buti et al., 2004; Fogeda et al., 2009a, 2009b; Maria Teresa Pérez-Gracia et al., 2007), demonstrating the zoonotic transmission of this genotype. Throughout the years HEV-3 has been the most identified genotype in Portugal and Spain, just like in other European countries, confirming genotype 3 as the most important cause of autochthonous hepatitis E in industrialized countries (Arends et al., 2014). Notwithstanding, other HEV genotypes, such as HEV-1 have been detected in the Iberian Peninsula (Maria Buti et al., 2004; Fogeda et al., 2009a, 2009b; Lens et al., 2015; Mesquita et al., 2018; Rodriguez-Manzano et al., 2010) but as expected, this genotype has been detected only in travelers or emigrants returning from HEV endemic regions (Maria Buti et al., 2004; Fogeda et al., 2009a, 2009b; Lens et al., 2015; Mesquita et al., 2018; Rodriguez-Manzano et al., 2010).

The potential role of HEV in patients with acute hepatitis non A-C was evaluated in a longitudinal nationwide study in Spain that concluded HEV was not the cause of acute hepatitis in most cases (Muñoz-Chimeno et al., 2022) however, in a study carried out in Barcelona it was found that HEV was the second leading cause of acute hepatitis (Li et al., 2013).

Studies focused on transplant recipients in the Iberian Peninsula have identified HEV-3 and also anti-HEV IgM/IgG in renal, liver, lung and other solid organ transplant recipients (Ambrocio et al., 2019; Breda et al., 2014; Carmo et al., 2016; Lens et al., 2015; Senosiain et al., 2016). Knowing that HEV-3 infection may progress into a chronic hepatitis in immunosuppressed patients (Wang & Meng, 2021), HEV diagnosis in these type of patients should be carried out since they can benefit from ribavirin treatment (Correia et al., 2022). HEV-3 has also been reported in HIV patients (Rivero-Juarez et al., 2017b), with some studies showing low or no detection rates of HEV RNA in these patients, (Jardi et al., 2012; Madejón et al., 2009; A. Rivero-Juarez et al., 2017b; Rodríguez-Tajes et al., 2020), in spite of the high anti-HEV seroprevalences detected in these patients (Filipe et al., 2022; López-López et al., 2019, 2022; Mateos-Lindemann et al., 2014; Merchante et al., 2015; Pineda et al., 2014; Riveiro-Barciela et al., 2014; Antonio Rivero-Juarez et al., 2015a, 2015b; Vázquez-Morón et al., 2019).

The studies carried out in the Iberian Peninsula in pregnant women never reported the presence of HEV RNA (Maria Buti et al., 2010; Lindemann et al., 2010), however HEV seropositivity has been described in this group (Maria Buti et al., 2010). This suggests that while the virus may not be actively replicating in pregnant women, they may have been exposed to the virus at some point in the past and have developed antibodies against it. It is important to continue studying the prevalence of HEV in pregnant women, as it can inform preventive measures to reduce the risk of transmission and potentially adverse outcomes in this vulnerable population.

Studies of HEV in animals (mainly pigs) started to proliferate in industrialized countries in the late 1990s following the first detection of HEV in pigs (Meng et al., 1997). In the Iberian Peninsula studies focused on HEV in animals, mainly swine, that also increased rapidly in the last two decades where the main circulating genotype is HEV-3. In Portugal the circulation of HEV-3 in swine has been observed in different instances with reports describing this genotype 3 in pigs and wild boars (Alessandra Berto et al., 2012a, 2012b; Mesquita et al., 2016a, 2016b, 2016c; Santos-Silva et al., 2023). More recently HEV-3, subgenotype 3e was identified in red deer (Moraes et al., 2022) and antibodies anti-HEV were detected in wild boars, wild rabbits and sheep from Portugal (Gonçalves et al., 2018; Lopes & Abrantes, 2020; Mesquita et al., 2020). Interestingly, the work that described the presence of IgG anti-HEV in sheep also suggested an increase of HEV infection in workers occupationally exposed to sheep (Mesquita et al., 2020). Likewise, many studies from Spain have confirmed the high circulating of HEV-3 in swine populations in all the country, in large or family flocks, and in all age groups with several studies reporting low to very high detection rates in pigs (Berto et al., 2012a, 2012b; Casas et al., 2011a, 2011b; Clemente-Casares et al., 2003; Deus et al., 2007; Deus et al., 2008a, 2008b; Deus et al., 2008a, 2008b; Fernández-Barredo et al., 2006, 2007; García et al., 2020; Kukielka et al., 2016; Lopez-Lopez et al., 2018; Martín et al., 2007; Navarro et al., 2020; Risalde et al., 2020; Riveiro-Barciela et al., 2015; Antonio Rivero-Juarez et al., 2020; Seminati et al., 2008). Concerning anti-HEV antibody detection, similar results have been observed when comparing them to HEV RNA in Spain (Casas et al., 2009, 2011a, 2011b; Clemente-Casares et al., 2003; Deus et al., 2008a, 2008b; García et al., 2020; Jiménez De Oya et al., 2011; Kukielka et al., 2016; Martín et al., 2007; Navarro et al., 2020; Peralta et al., 2009a, 2009b; Pina et al., 2000; Seminati et al., 2008). Interestingly, one study reported swine HEV-3 infection dynamics in a longitudinal survey conducted in farrow-to-finish farms showing the presence of HEV RNA in all stage groups (Casas et al., 2011a). This data is alarming since Spain is considered the biggest producer of pork meat and pork meat products in Europe (MAPA, 2021). HEV-3 circulation has also been observed in wild boars from Spain, with virus detection rate ranging from 1 to 37.9% (Caballero-Gómez et al., 2019a; de Deus et al., 2008a, 2008b; Kukielka et al., 2016; Navarro et al., 2020; Risalde et al., 2017, 2022; Antonio Rivero-Juarez et al., 2018c, 2020) and HEV seroprevalence varying between 5.2 and 62.8% (Barroso et al., 2021; Boadella et al., 2012; Caballero-Gómez et al., 2019a; Deus et al., 2008a, 2008b; Kukielka et al., 2016; Pina et al., 2000; María A. Risalde et al., 2017; Wang et al., 2019). In general, HEV-3 is highly prevalent in swine populations throughout the Iberian Peninsula posing a potential high risk of zoonotic transmission through the consumption of raw or undercooked pork meat or pork derived products.

The detection of HEV-3 has also been reported in other mammals of the Iberian Peninsula, namely in red deer (Mariana Boadella et al., 2010; Moraes et al., 2022), equines and Iberian lynx in which subgenotypes 3e–g were identified (Rivero-Juarez, et al., 2022; García-Bocanegra et al., 2019).

Serological studies performed in the Iberian Peninsula on different mammals species, have reported the presence of antibodies anti-HEV in rabbits, sheep, cows, goat, wild rodents, chicken, red deer, dogs, cats and lynx (Mariana Boadella et al., 2010; Caballero-Gómez et al., 2022a, 2022b, 2022c; Caballero-Gómez et al., 2022a, 2022b, 2022c; Rivero-Juarez, et al., 2022; Kukielka et al., 2016; Lopes & Abrantes, 2020; Mesquita et al., 2020; Peralta et al., 2009a, 2009b; Peralta et al., 2009a, 2009b). Antibodies anti-HEV have also been detected in rabbits in Japan (Mendoza et al., 2021) and in Germany (Hammerschmidt et al., 2017), in dogs in Switzerland (Veronesi et al., 2021), in sheep, cows, goat, wild rodents, chicken, dogs and cats in China (Li et al., 2013; Liang et al., 2014; Sun et al., 2016; Wu et al., 2015; Zhang et al., 2008), in red deer in Norway (Sacristán et al., 2021), however, the role of these animals as zoonotic reservoirs of HEV is still unclear.

Serological studies have also targeted non-human primates, with one study in the Iberian Peninsula reporting antibodies anti-HEV IgG in primates from zoos with a seroprevalence of 4.4%, being the first report of seropositivity in *Varecia variegata*, *Pan troglodytes*, and *Macaca sylvanus*, suggesting that these primates species of the *Hominidae* family are susceptible to HEV infection (Caballero-Gómez et al., 2019b). Non-human primates have been gaining interest in recent years. A Chinese study evaluated the infectivity of milk from HEV infected cow ingested by rhesus macaques, a species recognized for the high susceptibility to HEV infection (Huang et al., 2016). In Germany HEV seroprevalences have also been reported in non-human primates from zoos (Spahr et al., 2018) and in monkeys from Japan (Hirano et al., 2003).

Lastly, in the field of the environment, the Iberian Peninsula has also been a focus of a variety of studies on HEV, that began recently in Portugal, whereas in Spain started earlier, specifically in 1998. In Portugal, HEV-3 was detected in drinking water (Salvador et al., 2020), and in wastewater from treatment plants, where subgenotypes 3i and 3f were identified (Matos et al., 2018). In Spain, several studies have detected HEV in sewage (Albinana-Gimenez et al., 2006; Clemente-Casares et al., 2003, 2009; Pina et al., 1998, 2000; Randazzo et al., 2018; Rodriguez-Manzano et al., 2010; Rusiñol et al., 2015; Rusiñol et al., 2020a, 2020b). Specifically, genotypes 1 and 3 were identified suggesting that genotype 1 is also circulating, maybe imported from endemic regions, at least in low levels, in industrialized countries (Rodriguez-Manzano et al., 2010), and in manure (Fernández-Barredo et al., 2006, 2007; M. García et al., 2014). In influent wastewaters of four wastewater treatment plants HEV was detected (Cuevas-Ferrando et al., 2020), but not in effluent water, lagooning influent and effluent wastewater, reservoir water, river water, conventional activated sludge, wetland and coquina clams (Albinana-Gimenez et al., 2006; Bosch et al., 2001; Fernandez-Cassi et al., 2016; Rusiñol et al., 2020a, 2020b). The infectivity of HEV found in contaminated drinking water was confirmed in *Rhesus* monkey (Pina et al., 1998) and a similarity between the HEV-3 detected in pig slaughterhouse sewage and that detected in the serum of a human with acute hepatitis was demonstrated (Pina et al., 2000). Moreover, HEV-3 was detected in groundwater and raw sewage where porcine contamination was identified (Rusiñol et al., 2020a). Spain is the biggest producer of porcine products in Europe (MAPA, 2021). Given the zoonotic nature of HEV-3, contaminated streams impacted by swine have been found and reported in various countries (Kasorndorkbua et al., 2005; Santos et al., 2011). The absence of HEV-3 in any compost intended for sale as a soil fertilizer indicates that composting might be an effective means of removing HEV and decreasing the risk of transmission from pigs to people.

Some studies have also shown HEV in sea urchins, mussel and other kind of bivalve mollusks at high rates with zoonotic genotype 3 detected (Diez-Valcarce et al., 2012; João R. Mesquita et al., 2016c; Rivadulla et al., 2019; Santos-Ferreira et al., 2020). Criteria for the detection of enteric viruses in environmental samples are limited, so perhaps because of this fact, the difficulties in studying HEV in environmental samples are further exacerbated, highlighting a large discrepancy in results between studies. ISO 15216 provides proven molecular methodologies for the detection and quantification of hepatitis A virus and norovirus in high-risk food categories, such as bivalves mollusks (Lowther et al., 2019). However, these methods are not validated for the detection of other enteric viruses, such as HEV in foodstuffs, leading to an inability to ensure the safety of these products (Lowther et al., 2019).

As far as we know this is the first systematic review focusing on HEV studies in humans, animals and environment, the One Health triad, in the Iberian Peninsula. In short, by compiling all individual data of the triad, this approach expands the comprehensive information in a One Health perspective, enabling a focus on health problems such as food safety, zoonotic diseases, and environmental contamination.

## Conclusion

Overall, the present review shows the circulation of several HEV genotypes, namely HEV-1 to HEV-6, and *Rocahepevirus*, in humans, animals, and environment in the Iberian Peninsula. As expected for developed countries, HEV- 3 was the most common genotype circulating in humans in Portugal and Spain, having been identified in the majority of autochthonous cases, with HEV-1 been only detected in travelers and emigrants returning from HEV endemic regions. Being Spain a region with high circulation of HEV-3 and the country with the highest pork production in Europe and given the zoonotic transmission of this genotype by consumption of pork and pork products. In our view, it is important to establish a surveillance system for HEV in swine in the Iberian Peninsula, and to include HEV in the differential diagnosis of acute and chronic hepatitis in human patients, regardless of their travel history. Moreover, putting surveillance in place is necessary to fully understand the extent of this disease and the HEV strains circulating in the Iberian Peninsula and their potential public health hazards.

## Supplementary Information

Below is the link to the electronic supplementary material.

Supplementary file (DOCX 119 KB)

## Data Availability

The corresponding author can provide the data that was presented in this study upon request.
