# Supplementary material for: Hepatitis E Virus in the Iberian Peninsula: A Systematic Review
Source: Food Environ Virol. 2023 Jul 11;15(3):193–211. doi: 10.1007/s12560-023-09560-5 (PMC10499749; doi:10.1007/s12560-023-09560-5)
Supplement: Supplementary file 1 — Supplementary file (DOCX 119 KB) [file 12560_2023_9560_MOESM1_ESM.docx]

**Supplementary Table 1**. HEV molecular studies in human samples, Iberian Peninsula.

| **Country** | **Area of study** | **Sampling date** | **Population details** | **Sample type** | **Number HEV positive samples /total tested (%)** | **HEV RNA detection method** | **HEV Genome target region** | **HEV Genotype/ Subgenotype** | **Additional data** | **References** |
| --- | --- | --- | --- | --- | --- | --- | --- | --- | --- | --- |
| Portugal | Porto | 2012 | Man with acute hepatitis complicated by Guillain-Barre syndrome | Sera | NAp | Nested RT-PCR | ORF 1 | 3a | First report of neurological disorder associated with autochthonous HEV3 infection in Portugal | [25] |
|  | Lisbon | NA | Woman with severe acute hepatitis | Sera | NAp | RT-PCR | ORF2 | 3 | Hepatitis E that required hospitalization in a 58-year-old woman with concomitant signs of autoimmunity | [26] |
|  | Coimbra | 2012-2013 | Two renal transplant recipients with chronic hepatitis | Sera | NAp | RT-PCR | ORF1 | 3c | First report of chronic hepatitis E in transplant recipients in Portugal | [59] |
|  | Lisbon | NA | Liver transplant recipients with chronic hepatitis | NA | 5/141 (3.5%) | RT-PCR | NA | 3 | HEV prevalence in liver-transplant settings | [27] |
|  | NA | 2015 | Healthy child positive for IgM anti-HEV | Sera | NAp | RT-PCR | ORF1 | NAp | No HEV RNA was detected | [146] |
|  | NA | 2016 | Emigrants from an endemic areas (India) with severe acute hepatitis | Sera | NAp | RT-PCR | ORF 1 | 1 | First report in Portugal of imported cases of hepatitis E caused by genotype 1. Hospitalization was required given the severity of hepatitis | [30] |
|  | Lisbon | 2015 | Woman with severe acute hepatitis | Sera | NAp | Nested RT-PCR | ORF 1, 2 | 3a | Hepatitis E that required hospitalization in a 40-year-old woman with an autoimmune background (atrophic gastritis and Graves’ disease). An association between the severe hepatitis and the autoimmune background is suggested | [20] |
|  | Nationwide | 2015-2018 | Blood donors | Sera | 4/20,393 (0.02%) | RT-PCR | NA | ND | Incidence of HEV infection among blood donors were compared between Germany and Portugal, being 5x higher in Germany. Infections peaked in summer months and was significantly higher amongst males (p = 0.0002), but was not associated with ABO or Rh(D) blood group phenotypes | [31] |
|  | Lisbon | 2019 | Man with history of myelofibrosis | Sera | NAp | RT-qPCR | NA | ND | First case of HEV infection in a patient with myelofibrosis under ruxolitinib. Despite developing IgG anti-HEV the patient displayed continued viral replication until the suspension of ruxolitinib | [28] |
|  | Coimbra | 2014-2017 | Three kidney transplant (KT) recipients | Sera | NAp | RT-qPCR | NA | ND | Authors show the importance of HEV screening in KT recipients with abnormal liver function. None was able to clear the infection spontaneously. Treatment with ribavirin associated with reduction of immunosuppressive drugs was effective. | [160] |
|  | Porto | 2019 | HIV patients | Sera | 0/99 (0%) | RT-PCR | NA | NAp | No HEV RNA was detected. HEV detection was only performed in patients with HEV seroreactivity or if very immunosuppressed. | [29] |
| Spain | Barcelona | 1989-1999 | Patients with acute hepatitis | Sera | 3/37 (8.1%) | Nested RT-PCR | ORF 1, 2 | 6* | * The two HEV strains detected in Spanish patients were classified as genotype 6 in accordance to classification proposed by Schlauder et al., 1999 J Med Virol, 57 | [32] |
|  | Cádiz | 1999-2001 | Patients with acute hepatitis | Sera | 1/37 (2.7%) | Nested RT-PCR | ORF 2 | † | † The human strain detected had considerable homology with the sequences of HEV strains/isolates of European origin | [33] |
|  | Barcelona | 2003 | Patients with acute hepatitis | Sera | 3/11 (27.2%) | Semi-nested RT-PCR | ORF 1, 2 | 1, 3 | HEV genotype 1 positive patient was a traveller from an endemic area (Ethiopia) and two cases of genotype 3 were autochthonous | [35] |
|  | Madrid | NA | Slaughterhouse worker with acute hepatitis signs | Sera | NAp | Nested RT-PCR | ORF 2 | 3f | First report of autochthonous HEV infection in a slaughterhouse worker (the only risk factor seemed to be his occupation). | [34] |
|  | Nationwide | 2000 - 2008 | Patients with acute hepatitis | Sera | 13/95 (13.7%) | Nested RT-PCR | ORF 1, 2 | 1a 3f, 4 | 1a strains were from travellers from India and Bangladesh, and from an African immigrant.  3f strains were from patients who did not travel abroad recently (autochthonous).  The genotype 4 sequence was from a traveller returning from Vietnam. | [154] |
|  | Madrid | 2007-2008 | Acute hepatitis of unknown aetiology (HEV suspected) | Sera | 8/153 (5.22%) | RT-PCR | ORF 1, 2 | ND | Authors show the importance of differential diagnosis of hepatitis E virus, cytomegalovirus and Epstein-Barr virus infection in patients with IgM anti-HEV | [38] |
|  | Madrid | NA | HIV infected patients with low CD4 counts (a), HIV infected patients with chronic cryptogenic hepatitis (b), immigrants from risk area (c) | Sera | a- 0/50 (0%),  b - 0/43 (0%),  c - 0/33 (0%) | Nested RT-PCR | NA | NAp | HEV infection showed no relevant role in neither among HIV infected patients with advanced immunodeficiency or idiopathic liver enzyme elevations, nor in immigrants from HEV endemic regions. | [162] |
|  | Catalonia | 2004 | Pregnant women with high titters of IgG anti-HEV | Sera | 0/9 (0%) | Nested RT-PCR | ORF 1, 2 | NAp | Absence of HEV RNA despite of high titters of IgG anti-HEV | [71] |
|  | Barcelona | 2004-2007 | Patients with acute hepatitis | Sera | 4/19 (21%) | Nested RT-PCR, RT-qPCR | ORF 2 | 1, 3 | HEV genotype 1 was from a patient that had travelled to India in the previous month. Cases of genotype 3 were autochthonous | [72] |
|  | Madrid | 2007-2010 | Pregnant women with IgM anti-HEV | Sera | 0/7 (0%) | Nested RT-PCR | NA | NAp | Absence of HEV RNA despite presence of anti-HEV IgM | [40] |
|  | Valencia | NA | Pig workers (a), blood donors (b) | Sera | a – 0/113 (0%)  b – 0/99 (0%) | Nested RT-PCR | ORF 2 | NAp | Absence of HEV RNA despite high seroprevalence of anti-HEV IgG. | [74] |
|  | Madrid | NA | Woman with fulminant hepatic failure | Sera | NAp | Nested RT-PCR | ORF 2 | 3f | First report of autochthonous fulminant hepatitis E in a European non-pregnant women taking oral contraceptives. Authors suggest oral contraceptives as a risk factor since it mimics the hormonal status of the pregnancy. | [40] |
|  | Catalonia | 2011 | HIV infected patients positive for anti-HEV | Sera | 3/22 (13.63%) | RT-PCR | NA | 3 | HEV was detected in 3 patients: HCV associated liver cirrhosis (n=2); without chronic liver disease (n=1). None presented clinical symptoms or biochemical data suggestive of acute hepatitis. | [161] |
|  | Madrid | 2011 - 2012 | HIV infected patients positive for anti-HEV | Sera | 1/18 (5.55%) | Nested RT-PCR | NA | ND | Presence of HEV RNA in the absence of clinical symptoms related to viral acute hepatitis (ALT and AST also normal). | [37] |
|  | Madrid, Seville | 2008 - 2009 | Chronic hepatitis E in HIV infected patients with severe immunosuppression | Sera | NAp | Nested RT-PCR | NA | ND | First report showing that monotherapy with RBV may be transiently efficacious against chronic hepatitis E in HIV infected patients | [41] |
|  | Andalusia | 2009-2011 | HIV infected patients | Sera | 1/161 (0.6%) | RT-qPCR | NA | ND | In this follow up study less than 1% of HEV-seropositive individuals tested positive for serum HEV RNA at baseline and no patient evolved to chronic HEV infection after seroconversion | [164] |
|  | Madrid | NA | HIV infected patients (representative group) | Sera | 1/45 (2.2%) | Nested RT-PCR | ORF 2 | ND | HEV RNA was only searched in IgG anti-HEV positive patients. Authors suggest that chronic coinfection HEV-HIV seems to be a very rare event. | [75] |
|  | Andalusia | 2006 - 2008 | HIV infected patients with unexplained elevations of liver stiffness (a), HIV infected patients with normal liver stiffness (b) | Sera | a – 1/31 (3.2%)  b – 0/31 (0%) | RT-qPCR | NA | ND | HEV RNA was detected in only one patient with a documented chronic hepatitis E with progression to cirrhosis. | [166] |
|  | Barcelona | 2011-2013 | Patients from referral centre (HEV infection suspected) | Sera | 10/392 (2.5%) | Nested RT-PCR | ORF2 | 1, 3 | HEV RNA detected in 10 patients: travellers from endemic areas (n=4); solid organ transplant recipients (n=5); immunocompetent patient with agranulocytosis (n=1) that resulted in a fatal outcome (first report of this kind). Genotype 1 found only in the travellers. | [156] |
|  | Barcelona | 2012 | Immunocompromised patient (Waldeström macroglobulinemia with immunoglobulin A deficiency) with severe acute hepatitis E | Sera | NAp | RT-qPCR | ORF 2 | 3f | Hepatitis E transmitted by pork meat ingestion demonstrated by phylogenetic analysis. | [91] |
|  | Barcelona | 2013 | Blood donors | Sera | 3/9,998 (0.03%) | RT-PCR | ORF 3 | 3f | The positive blood donors showed normal ALT. HEV RNA positivity rate was one per 3333 donations. | [47] |
|  | Madrid | 2014 | HIV infected patients (a), HCV infected patients (b), and HIV/HCV infected patients (c), healthy patients (d) | Sera | 1/11 (9%) | RT-PCR | NA | ND | HEV RNA was only searched in IgM positive samples. The only patient with HEV RNA was HIV-HCV coinfected. | [76] |
|  | Madrid | NA | Immunocompromised patients with inflammatory bowel disease | Sera | 0/46 (0%) | Nested RT-PCR | NA | NAp | HEV RNA was searched randomly and only in IgM/IgG anti-HEV positive patients. No chronic infection by HEV was detected. | [157] |
|  | Leon | 2008-2014 | Patients with acute hepatitis E | Sera | 10/23 (43.9%) | Nested RT-PCR | ORF 1, 2 | ND | The most of cases (N = 22) were autochthonous but one was associated with travel (China). | [77] |
|  | Barcelona | 2015 | Immunocompetent with acute hepatitis E | Sera | NAp | RT-qPCR | ORF2 | 3 | First report in Europe of acute full-blown hepatitis E in an immunocompetent linked to the transfusion of red blood cells, confirmed by phylogenetic analysis. History of tuberculosis treatment and abnormal liver function. Received red blood cell transfusion 2 months previously (due to aneurysm surgery). | [49] |
|  | Andalusia | 2015 | HIV infected patients with acute hepatitis and family members | Sera | NAp | RT-qPCR | ORF2 | 3 | Familial hepatitis E outbreak linked to wild boar meat consumption, confirmed by phylogenetic analysis (sequences of humans and meat showed 100% homology). | [51] |
|  | Cordoba | NA | Patients with HIV/HCV coinfection | Sera | NAp | RT-qPCR | ORF2 | 3 | Two cases of acute symptomatic HEV genotype 3 infection in patients carrying anti-HEV IgG, after successful completion of HCV therapy. HEV reactivation or reinfection was questioned. | [44] |
|  | Cordoba | 2012-2016 | Liver allografts from donors (a), transplant recipients (b) | Liver | a- 1/105 (0.95%), b-0/105 (0%) | RT-PCR | NA | 3f | Authors suggest a potential risk of HEV transmission through liver allograft transplants | [52] |
|  | Cordoba | 2016-2017 | Patients with acute hepatitis (a), blood donors (b) | Saliva, sera | Saliva:  a – 8/34 (23.5%), b -– 0/12 (0%),  Sera:  a - 8/34 (23.5%),  b – 0/12 (0%) | RT-qPCR | NA | ND | Authors suggest that acute HEV infection could be diagnosed by assessing viral load in saliva. | [53] |
|  | Málaga | 2016 | Patient with fulminant hepatic failure due to HEV | NA | NAp | RT-PCR | NA | NAp | No HEV RNA was detected despite anti-HEV IgM and IgG were positive. | [79] |
|  | Barcelona | 2017 | Patient with acute hepatitis E after receiving plasmaphoresis for treatment of thrombotic thrombocytopenic purpura | Sera | NAp | RT-qPCR | ORF2 | 3f | First report of acute HEV infection transmitted by cryosupernatant plasma used for plasmapheresis (confirmed by phylogenetic analysis).  One cryosupernatant plasma among 99 different blood components used for the patient’s plasmapheresis was positive for HEV RNA (viral load of 5000 to 10,000 IU/mL). | [45] |
|  | Barcelona | 2018 | Patient with acute hepatitis and metabolic syndrome | Sera | NAp | NA | NA | 3 | Acute hepatitis E can trigger rapid liver impairment and clinical decompensation in patients with underlying liver disease. | [36] |
|  | Galicia | NA | Patient with acute hepatitis and neurological symptoms | Sera, cerebrospinal fluid | NAp | Nested RT-PCR | ORF 2 | 3f | First report in Spain of a simultaneous acute meningitis and hepatitis, secondary to autochthonous HEV infection in an immunocompetent. HEV RNA detected in serum and cerebrospinal fluid. Authors suggest HEV infection as a cause of meningitis. | [50] |
|  | Madrid | 2010-2017 | Three lung transplant recipients with symptoms and alterations in liver enzymes | NA | NAp | RT-PCR | NA | ND | One patient (not treated/ribavirin) died of progressive hepatic disease (post-mortem diagnosis of HEV infection complications). | [158] |
|  | Nationwide | 2012-2014 | HIV/HCV infected patients (a), HIV infected patients (b), healthy population (c) | Sera | a – 0/44 (0%)  b – 0/6 (0%)  c – 0/2 (0%) | Nested RT-PCR | ORF 1 | NAp | Neither HEV-RNA was detected nor any significant differences between groups was observed. | [169] |
|  | Ciudad Real | 2017-2018 | Blood donors | Sera | 4/11,313 (0.035%) | Nested RT-PCR | ORF 2 | 3 | A high prevalence of HEV infection in blood donors from south- central Spain was found with a ratio of one positive donation per 2828 donations.  Five patients received transfusions from HEV- positive blood donations but none of them showed an increase in alanine aminotransferase levels and HEV RNA after transfusion. | [48] |
|  | Santiago de Compostela | 2018-2019 | Two transplant recipient patients | Sera | NAp | RT-qPCR | ORF2 | 3f | Authors show that the use of ribavirin (12 weeks) for the treatment of HEV infection in organ transplant patients is effective. | [46] |
|  | Cordoba | 2012-2014 | HIV infected patients according to PROGINS: homozygous wild-type (a), heterozygous (b), homozygous (c) | Sera | RNA and/or IgM  a – 26/39 (66.7%)  b – 8/39 (20.5%)  c – 5/39 (12.8%) | RT-PCR | NA | ND | None of PROGINS homozygous with recent HEV infection (RNA and/or IgM) presented symptoms. Authors concluded that PROGINS mutation plays a protective role against HEV infection. | [42] |
|  | Zaragoza | 2011-2018 | Two immunocompetent patients with Parsonage-Turner syndrome (with clinical/epidemiological suspicion of hepatitis E) | Sera | NAp | RT-PCR | ORF2 | 3f | HEV should be considered in patients with neuralgic amyotrophy, including those with the absence of liver cytolysis. | [18] |
|  | Barcelona | 2012 | Liver-kidney transplant patient with chronic hepatitis E | Sera | NAp | RT-PCR | NA | 3 | Failure of sofosbuvir plus ribavirin therapy, evolving to death | [15] |
|  | Barcelona | 2014-2018 | Patient with viral acute hepatitis | Sera | RNA and/or IgM  18/100(18%) | RT-qPCR | NA | ND | HEV was the second most common causes of acute hepatitis. Four of the 18 (22%) acute HEV infections occurred in immigrants. No relationship was found between immigrant status or sexual risk behaviour and acute HEV infection (RNA and/or IgM). | [80] |
|  | Barcelona | 2014-2018 | Patients with chronic autoimmune hepatitis (a), acute autoimmune hepatitis (b), acute non-autoimmune hepatitis (c) | Sera | a - 0/50 (0%)  b - 0/30 (0%)  c - 1/25 (4%) | RT-qPCR | NA | ND | None of the patients with autoimmune hepatitis tested positive for HEV-RNA, being only detected in a patient with acute non-autoimmune hepatitis. | [150] |
|  | Catalonia | 2014-2019 | Patients with self-limited acute hepatitis E (a), HEV RNA-positive blood donors (b) | Sera | Baseline:  a – 7/17 (41%)  b – 50/50 (100%)  End of follow up (34 mo):  a – 0/25 (0%)  b – 0/50 (0%) | RT-qPCR | NA | ND | The inclusion of HEV RNA-positive donors was used to obtain data on predictive values of the anti-HEV IgM and HEV antigen assays used in diagnostic of acute HEV infection. HEV antigen showed better performance in terms of diagnostic accuracy and could be useful for diagnosing acute HEV infection in settings where HEV RNA is not available. | [43] |
|  | Nationwide | 2016-2018 | Patients with drug-induced liver injury (DILI) tested positive for anti-HEV IgM | Sera | 2/8 (25%) | RT-PCR | NA | NAp | A search for active HEV infection is advised in patients assessed or suspicion of DILI. Underlying hepatic diseases (p < 0.001) and AST peak >20 fold upper limit of normal (p = 0.002) were associated with the diagnosis of acute hepatitis E. | [54] |
|  | Barcelona | 2017-2018 | MSM (a) and non-MSM (b) with acute hepatitis A | Sera | a – 0/59 (0%)  b – 0/26 (0%) | Nested RT-PCR | ORF 2 | NAp | This study suggests that MSM are not at a higher risk for HEV infection. The lack of detectable HEV‐RNA lead authors to concluded that HEV does not spread by sexual contact or person‐to‐person. | [163] |
|  | Nationwide | 2009-2019 | Patients with hepatitis non A-C | Sera | 409/5,197 (7.87%) | Nested RT-PCR | ORF 2 | 3c, 3f, 3m | This study describes the distribution of the HEV-3 subtypes in Spain at national level between 2009 and 2019. The dominant subtype (88.3%) was HEV-3f (subclusters HEV-3f-B, HEV-3f-A1 and HEV-3f-A2), the second most common subtype (7%) was HEV-3m (subclusters HEV-3m-A and HEV-3m-B) and the third (4.1%) was HEV-3c. The subtype HEV-3f was widely distributed throughout Spain but was most frequent in the north (p = 0.000), HEV-3c was most prevalent in the east (p = 0.005), HEV-3m was reported in the centre, north and south of Spain, but no cases were found in the east. | [39] |
|  | Catalonia | 2017-2020 | Blood donors | Sera | 151/655,523 (0.023%) | RT-qPCR  RT-PCR | ORF 3  ORF 2 | 3c, 3f | No cases of HEV-transfusion-transmitted infection were reported during these 2.5 years of HEV RNA universal screening study in Catalonia. Most HEV-infected donors were asymptomatic and spontaneously resolved the infection. They reported consumption of pates and sausages | [17] |
|  | Cordoba | 2012-2020 | HIV/HCV-coinfected cirrhotic patients | Sera | Baseline  0/75 (0%)  Follow up  0/75 (0) | RT-qPCR | ORF 3 | NAp | Follow-up of 5.1 years was performed.  None of the patients showed detectable HEV viral load during the study period. | [81] |

NAp - not applicable; NA - not available; ND – Not determined; MSM – Men who have sex with man

**Supplementary Table 2.** HEV serological studies in human samples, Iberian Peninsula.

| **Country** | **Area of study** | **Sampling date** | **Population details** | **Number anti -HEV positive samples /total tested (%)** | **HEV serological assay** | **Additional data** | **References** |
| --- | --- | --- | --- | --- | --- | --- | --- |
| Portugal | Porto | NA | Haemophiliacs (a), blood donors (b) | IgG  a - 2/52 (3.8%)  b - 11/341 (3.2%) | IgG anti-HEV (ELISA^1^) | No increased risk of HEV transmission by transfusion (p>0.05). | [55] |
|  | North | NA | Blood donors | IgG 37/1473 (2.5%) | IgG anti-HEV  (ELISA^1^) | Seroprevalence in blood donors from North Portugal. No difference was observed regarding sex, regions or presence of antibodies to HBV. | [56] |
|  | North | NA | Blood donors (a), patients with chronic hepatitis (b) | IgG  a - 2/50 (4%)  b - 7/103 (6.8%) | IgG anti-HEV  (ELISA^1^) | First assessment of HEV seroprevalence in the north of Portugal. Seropositivity was not significantly higher in chronic liver disease patients compared to blood donors. | [142] |
|  | Coimbra | 2010 | Patient with acute hepatitis | IgM positive  IgG positive | IgM / IgG anti-HEV (ELISA^5^) | First report in Portugal of an autochthonous case of acute hepatitis E (not confirmed by RNA HEV in sera). | [57] |
|  | North | 1993 | Mothers (a) and their newborns (b) | IgG  a - 4/12 (33.3%)  b - 4/12 (33.3%) | IgG anti-HEV  (ELISA^4^) | An efficient transplacental transport of anti-HEV IgG was demonstrated as well as circulation of autochthonous HEV in Portugal in the early 1990s. | [58] |
|  | Porto | 2012 | Man with acute hepatitis complicated by Guillain-Barre syndrome | IgM positive  IgG negative | NA | First report of an autochthonous acute hepatitis E complicated by Guillain-Barre syndrome. | [25] |
|  | Lisbon | NA | Woman with severe acute hepatitis with concomitant signs of autoimmunity | IgM positive  IgG negative | IgM/IgG anti-HEV (ELISA^5^) | Hepatitis E that required hospitalization in a 58-year-old woman with (confirmed by RNA HEV in sera). | [26] |
|  | Coimbra | 2012-2013 | Two renal transplant recipients with chronic hepatitis (a, b) | IgM  a – positive  b – positive  IgG  a – positive  b – negative | IgM / IgG anti-HEV (ELISA^6^) | First report of chronic hepatitis E in transplant recipients in Portugal (confirmed by RNA HEV in sera). | [59] |
|  | Nationwide | 2013 | Pet veterinarians (a), general population (b) | IgG  a - 36/373 (9.7%)  b - 16/120 (13.3%) | IgG anti-HEV  (ELISA^4^) | Pet veterinarians have no increased risk of HEV infection compared to the general population (*p*=0.231). | [62] |
|  | Nationwide | 1992-1995 | Children (1-13 years) | IgM 1/71 (1.4%)  IgG 2/71 (2.8%) | IgM/ IgG anti-HEV (ELISA^2^, immunoblot^3^) | First report showing evidence of autochthonous HEV infection in a paediatric population in the early 1990s. | [60] |
|  | Centre, North | 2015 | Workers occupationally exposed to swine (a), general population (b) | IgG  a - 35/114 (30.7%)  b - 160/804 (19.9%) | IgG anti-HEV  (ELISA^2^) | First report on the seroprevalence in workers occupationally exposed to swine from Portugal. An increased risk of HEV infection compared to the general population (*p*=0.002) was shown. | [63] |
|  | Nationwide | 2015 | Children aged  0-4 (a), 5-9 (b), 10-14 (c), 15-19 (d) | Total IgM 1/352 (0.28%)  a- 0/86 (0%)  b - 0/86 (0%)  c - 1/95 (1.1%)  d - 0/89 (0%)  Total IgG 4/352 (1.1%)  a - 1/86 (1.1%)  b - 0/86 (0%)  c - 2/95 (2.2%)  d - 1/89 (1.1%) | IgM/IgG anti-HEV  (ELISA^2^, immunoblot^3^) | This is the first national study of HEV seroprevalence in Portuguese children. No statistically significant difference was observed in HEV seroprevalence regarding gender, age group and region of residence. Authors also compared anti-IgG seropositivity of 2015 children cohort with a 1995 cohort matched by sex, age (1-13 years) and region. A significant decrease from the 1995 to the 2015 cohorts was found (7.0% to 0.9%; *p*=0.024). | [146] |
|  | Nationwide | 2015-2016 | General population aged  0–9 (a), 10–19 (b), 20–29 (c), 30–39 (d), 40–49 (e), 50–59 (f), 60–69 (g), 70+ (h) | Total IgM 8/1656 (0.48%)  a- 0/164 (0%)  b - 0/177 (0%)  c - 0/184 (0%)  d- 1/225 (0.4%)  e - 3/220 (1.4%)  f - 1/206 (0.5%)  g - 0/184 (0%)  h - 3/296 (1.0%)  Total IgG 270/1656 (16.3%)  a - 1/164 (0.6%)  b - 3/177 (1.7%)  c - 5/184 (2.7%)  d - 29/225 (12.9%)  e - 42/220 (19.1%)  f - 53/206 (25.7%)  g -48/184 (26.1%)  h - 89/296 (30.1%) | IgM/IgG anti-HEV (ELISA^2^) | Seropositivity increase significantly (p < 0.05) with age reaching its maximum in elder groups (0.6% in the 0–9 years group to 30.1% in people ≥ than 70 years); no difference between genders was observed. The seroprevalence also varied geographically (from 8 to 28%) with generally higher seropositivities in the most rural areas of Portugal, but difference was not statistically significant (*p*> 0.05). | [61] |
|  | Centre | 2017 | Workers occupationally exposed to ovine (a), general population (b) | IgG  a - 27/96 (29.3%)  b - 31/192 (16.1%) | IgG anti-HEV  (ELISA^2^) | First report on the seroprevalence in workers occupationally exposed to ovine from Portugal. An increased risk of HEV infection compared to the general population (*p*=0198) was shown. | [64] |
|  | Lisbon | 2019 | Patient with acute hepatitis | IgM positive  IgG positive | IgM/IgG anti-HEV (ELISA, market company not available) | First case of HEV infection in a patient with myelofibrosis under ruxolitinib. Despite developing IgG anti-HEV the patient displayed continued viral replication until the suspension of ruxolitinib | [28] |
|  | Coimbra | 2014-2017 | Three kidney transplant (KT) recipients | IgM positive  IgG positive | IgM/IgG anti-HEV (ELISA, market company not available) | Authors show the importance of HEV screening in KT recipients with abnormal liver function. None was able to clear the infection spontaneously. Treatment with ribavirin associated with reduction of immunosuppressive drugs was effective. | [160] |
|  | Porto | 2019 | HIV Patients | IgG  76/299 (25.4%) | IgG anti-HEV  (ELISA^4^) | The seroprevalence in this HIV cohort of patients was higher (25.4%) than that found in general population in the same region (18.1%). | [29] |
| Spain | Barcelona | 1989-1991 | Patients with acute hepatitis | IgM 0/341 (0%)  IgG 0/341 (0%) | IgM/IgG anti-HEV (ELISA^7^) | The prevalence of hepatitis viruses in acute sporadic hepatitis in Spain was evaluated. No case of HEV infection was detected. | [65] |
|  | Barcelona | 1990-1993 | Patients with acute hepatitis A (a), with acute hepatitis B/D (b), with acute hepatitis non-A-C (c), healthy subjects (d), haemophiliacs (e), patients on haemodialysis (f) | IgM  a – 0/244 (0%)  b – 2/48 (4.16%)  c – 0/90 (0%)  IgG  a – 10/244 (4%)  b – 3/48 (6.5%)  c – 5/90 (5.6%)  d – 3/54 (5.5%)  e – 0/55 (0%)  f – 3/50 (6%) | IgM/ IgG anti-HEV (ELISA^1^) | Acute hepatitis E is present in Spain. It accounts for 2.2% of acute non-A, non-B hepatitis and for 1% of all hepatitis cases. Authors concluded that HEV is a minor cause of acute hepatitis in  Spain. | [141] |
|  | Barcelona | NA | Haemophilic patients treated with clotting-factor concentrates (a), healthy individuals (b) | IgG  a – 0/22 (0%)  b – 0/22 (0%) | IgG anti-HEV  (ELISA^1^) | Clotting-factor concentrates seem to be safe against HEV. | [82] |
|  | Caceres | 1993-1994 | Rural child population (13-15 years) | IgG 5/95 (5.3%) | IgG Anti-HEV ^1^ | HEV prevalence was higher than other European countries | [66] |
|  | Madrid | NA | Blood donors (a), asymptomatic haemodialyzed patients (b) | IgG  a – 4/63 (6.3%)  b – 25/863 (2.8%) | IgG anti-HEV  (ELISA^1^) | Authors concluded (i) HEV incidence in Spain is similar to that in other Western European countries based on anti-HEV IgG data in blood donors; (ii) HEV infection must be considered in haemodialysis patients. | [67] |
|  | Madrid | NA | Blood donors (a), asymptomatic haemodialyzed patients (b), children with post-transfusion HCV infection(c) | IgM  a – 0/25 (0%)  b – 0/4 (0%)  IgG  a – 25/863 (2.8%)  b – 4/63 (6.3%)  c – 0/42 (0%) | IgM/IgG anti-HEV  (ELISA^1^, immunonblot^11^) | Authors concluded (i) HEV incidence in Spain is similar to that in other Western European countries based on anti-HEV IgG data in blood donors; (ii) HEV infection must be considered in haemodialysis patients; (iii) parenteral transmission of HEV to children is rare. | [144] |
|  | Barcelona | 1989-1999 | Patients with acute hepatitis | IgG 8/37 (21.6%) | IgG anti-HEV (ELISA)^1)^ | HEV was studied as a causal agent of acute hepatitis cases in the Spanish population. Anti-HEV antibodies were searched only among non-B non-C acute hepatitis patients. | [32] |
|  | Madrid | NA | Patients on peritoneal dialysis | IgM 0/4 (0%)  IgG 4/40 (10%) | IgM/IgG anti-HEV (ELISA^1,7^) | HEV prevalence in these patients was higher than that found in healthy people in Spain | [149] |
|  | Madrid | NA | Blood donors (a), immigrants from sub-Saharan Africa (b) | IgM  a – 0/25 (0%)  b- - 0/5 (0%)  IgG  a – 25/863 (2.9%)  b – 5/90 (5.5%) | IgM/IgG anti-HEV (ELISA^1,7^), immunonblot^11^) | Seroprevalence was 1.9 times higher in immigrants than in blood donors. For authors sub-Saharan immigrants cannot be considered a major risk for the transmission of HEV in Spain, based on data. | [68] |
|  | Cádiz | 1999-2001 | Patients with acute hepatitis | IgM 30/336 (8.92%) | IgM anti-HEV  (ELISA^8^, immunoblot^9^) | Only 7.7% of sera were positive for anti-HEV IgM by immunoblot, and amongst them, one serum was viremic. Author suggesting a correlation between viraemia and the reactivity in the immunoblot band of ORF2. | [33] |
|  | Madrid | 1999-2005 | Patients with sporadic autochthonous hepatitis E (a), imported hepatitis E cases (b) | IgM  a – 11/12 (91.6%)  b – 6/6 (100%)  IgG  a – 12/12 (100%)  b – 6/6 (100%) | IgM/IgG anti-HEV  (ELISA^8^, immunoblot^10^) | Authors concluded that HEV diagnosis should be suspected in patients with acute hepatitis and anti-HEV antibodies in serum without known risk factors such as traveling to endemic areas. | [73] |
|  | Calatonia | 2002 | General population (15 – 74 years) | IgG 96/1,280 (7.3%) | IgG anti-HEV  (ELISA^8^) | This is the first large Spanish study of the HEV seroprevalence. It was greater among males than among women (7.8% vs. 7%), increased with age for both sexes, from 3% among 15 - 24 years to 12% among >65 years of age. An association between HEV seroprevalence and subjects with a history minor surgery was observed. | [69] |
|  | Barcelona | NA | General population aged 16-30 (a), 31-40 (b), 41-50 (c), 51-60 (d) | IgG  Total 35/103 (34%)  a – 1/14 (7.1%)  b – 5/20 (25%)  c – 8/26 (30.8%)  d - 10/27 (37%) | IgG anti-HEV^14^ | Seroprevalence increased with age. Very low rate of seropositivity in the 16-30-year-old group positivity compared with other ages groups. | [147] |
|  | Valladolid | 2003 | General population aged <20 (a), 20-39 (b), 40-54 (c), ≥55 (d) | IgG  Total 3/365 (0.8%)  a - 0%  b - 0%  c - 1.4%  d - 1.9% | IgG anti-HEV (ELISA^11^) | Only one case among the three HEV seropositive had a history of a prior trip to a HEV-endemic area. | [148] |
|  | Madrid | NA | Slaughterhouse worker with acute hepatitis signs | IgM positive  IgG positive | IgM/IgG anti-HEV  (ELISA^8^, immunoblot^10^ ) | First report of autochthonous HEV infection (confirmed by RNA HEV in sera) in a slaughterhouse worker (the only risk factor seemed to be his occupation). | [34] |
|  | Calatonia | 2001 | Children (6-15 years) | IgM 2/57 (3.5%)  IgG 57/1,249 (4.6%) | IgM/IgG anti-HEV  (ELISA^8^) | This is the first large study analysing HEV seroprevalence in healthy children in Spain. Authors suggests an exposition of HEV in early childhood. Anti-HEV slightly decreased with age, from 4.6% in children aged 6 to 9 years to 3.8% in children aged 13 to 15 years (*p* = 0.4), was higher in girls than in boys (5.8% vs. 3.4%; *p* = 0.6), was higher in children living in urban than in rural areas (5.0% vs. 3.1%), in those not born and born in Spain (12.5% vs. 4.4%), and those in social classes IV and V (5.8%) compared those in social classes I to III (4.4%), although the differences were not statistically significant. | [70] |
|  | Madrid | 2000-2008 | Patients with acute hepatitis E and positive for HEV RNA | IgM  10/13 (76.9%)  IgG  9/13 (69.2%) | IgM/IgG anti-HEV (ELISA and immunoblot^10^ | Lack of detectable levels of both anti-HEV IgG and IgM in three HEV RNA positive patients that might be due to the sampling during the window period. | [154] |
|  | Madrid | 2007-2008 | Acute hepatitis of unknown aetiology (HEV suspected) (a), infectious mononucleosis (no liver involvement) (b) | IgM  a – 15/153 (9.8%)  b – 6/18 (33.3%) | IgM/IgG anti-HEV (ELISA^8,12,16^, immunoblot^10^) | Acute infection by EBV or CMV may induce false reactivity to anti-HEV IgM | [38] |
|  | Catalonia | 2004 | Pregnant women aged 15-24 (a). 25-29 (b), 30-34 (c), 35-49 (d) | IgM  a – 0/16 (0%)  b – 0/19 (0%)  c – 0/35 (0%)  d – 0/12 (0%)  IgG  Total 82/1,517 (5.4%)  a – 16/294 (5.4%)  b – 19/386 (4.9%)  c – 35/534 (6.6%)  d – 12/303 (4%) | IgM/IgG anti-HEV (ELISA^8^) | Seroprevalence varied according to age and was not associated with the place of residence (urban or rural habitat), educational level (primary education or not), social class (I–III and IV–VI) or place of birth. IgM was not found in any of the IgG positive samples tested. No case of acute hepatitis E was found during pregnancy, leading authors to suggest that HEV infection is exceptional in Spanish pregnant women. | [71] |
|  | Barcelona | 2004-2007 | Patients with acute hepatitis | IgG  19/19 (100%) | IgG anti-HEV  (ELISA^8^) | Four of the 19 analysed serum samples analysed were positive for HEV RNA, confirming that HEV circulates in the population and that it causes sporadic clinical cases of acute hepatitis. | [72] |
|  | Madrid | NA | Woman with fulminant hepatic failure | IgM positive  IgG positive | IgM/IgG anti-HEV  (ELISA^8^, immunoblot^10^) | First report of autochthonous fulminant hepatitis E (confirmed by RNA HEV in sera) in a European non-pregnant woman taking oral contraceptives. Authors suggest this treatment as a risk factor since it mimics the hormonal status of the pregnancy. | [40] |
|  | Valencia | NA | Pig workers (a), blood donors (b) | IgM  a – 0/113 (0%)  b – 0/99 (0%)  IgG  a – 21/113 (18.6%)  b – 4/99 (4%) | IgM/IgG anti-HEV  (ELISA^15^; Immunoblot^10^ ) | High seroprevalence in pig workers compared with unexposed population, supporting HEV infection as an occupational disease. Other risk factors were evaluated (travelled out of Spain, consumption of raw shellfish, raw vegetables and untreated water). Drank untreated water was also risk factor. | [74] |
|  | Cordoba | 2009-2011 | HIV infected patients | IgG  161/613 (26%) | IgG anti-HEV (ELISA^4^) | Rate of exposure to HEV among HIV infected patients is showed to be very high. HEV exposure more common in men than in women (28% vs. 18%; *p*=0.022) and increased with age: 16% (≤ 40 years), 26% (40-49 years) and 44% (>50 years), (*p*=0.000002). Seroreversion was detected in 19% of the HEV-seropositive patients at baseline (seroreversion was more common in patients with CD4+ below 500 cells/ml than those who remained seropositive, 77% vs. 46%; *p*=0.004). Most cases of acute HEV infection goes unnoticed. Evolution to chronic infection is extremely unusual. | [164] |
|  | Barcelona | 2011 | Patients with chronic liver disease without cirrhosis (a), chronic liver disease with cirrhosis (b), HIV infected patients without liver disease (c), HIV infected patients with liver disease (d), liver transplant without cirrhosis (e), liver transplant with cirrhosis (f), kidney transplant (g), healthy population (h) | IgG  a – 5/244 (2%)  b – 10/57 (17.5%)  c – 8/98 (8.2%)  d – 14/140 (10%)  e – 23/310 (7.4%)  f – 9/28 (32.1%)  g – 11/296 (3.7%)  h – 7/200 (3.5%) | IgG anti-HEV  (ELISA^8^) | A strong association between HEV seroprevalence and liver cirrhosis was found. Seroprevalence in patients with chronic liver disease with cirrhosis was higher than those without cirrhosis (17.5% vs 2%, p<0.01) and was also higher in liver transplant who developed cirrhosis than in liver transplant without cirrhosis (32.1% vs. 7.4%, p<0.01). Cirrhosis, liver transplantation and HIV infection were found to be a risk factors associated with HEV infection. | [165] |
|  | Madrid | NA | HIV infected patients (representative group) | IgM 3/45 (6.6%)  IgG 45/448 (10.4%) | IgM/IgG anti-HEV (ELISA^12^, immunoblot^10^ ) | IgM only search in IgG anti-HEV positive patients. A high seroprevalence of anti-HEV IgG antibodies was found and authors concluded that HIV infected patients can be considered a risk group for HEV infection. No difference was observed in seroprevalence regarding age, gender and CD4 count. Asymptomatic HEV infections detected in this group of HIV infected patients. | [75] |
|  | Andalusia | 2006 - 2008 | HIV infected patients with unexplained elevations of liver stiffness (a), HIV infected patients with normal liver stiffness (b) | IgG  a - 9/31 (29%)  b - 5/31 (16%) | IgG anti-HEV (ELISA^2,4^, immunoblot^3^) | Authors concluded that HEV infection seems to be more frequent among HIV infected patients with unexplained liver disease and it may be involved in the development of the latter disorder. | [166] |
|  | Barcelona | 2011-2013 | Patients attended in a referral centre with suspected HEV infection (acute or chronic) | IgG 23/392 (6%) | IgG anti-HEV  (ELISA^2^) | During the three-year period of this study 23 patients presented anti-HEV antibodies, including travellers from endemic areas, solid organ transplant recipients and an immunocompetent patient with agranulocytosis that resulted in a fatal outcome (first report of this kind). | [156] |
|  | Barcelona | 2012 | Immunocompromised patient (Waldeström macroglobulinemia with immunoglobulin A deficiency) with severe acute hepatitis E | IgM positive | NA | First reported case by zoonotic transmission after pork meat ingestion (confirmed by RNA HEV in sera). | [91] |
|  | Cordoba | 2012 - 2013 | HIV infected patients with low CD4+ cells (200 cells/ml) | IgM  1/115 (0.8%)  IgG  29/115 (25.2%) | IgM/IgG anti-HEV  (ELISA^4^) | HEV RNA was not detected among HEV seronegative patients. For that, authors suggest that HEV serological screening can be applied in HIV infected patients with low CD4+ cells, without compromising its diagnostic value. | [168] |
|  | Cordoba | 2012 – 2013 | HIV infected patients (a), HIV/HCV infected patients (b), HIV/HBV infected patients c), HIV/HCV/HBV infected patients (d) | Anti-HEV  Total 88/894 (9.8%)  a – 40/389 (10.03%)  b – 44/462 (9.5%)  c – 2/12 (16.6%)  d – 2/21 (9.5%) | IgM/IgG anti-HEV  (ELISA^4^; Immunoblot^3^) | No difference in seroprevalence was found when HIV monoinfected patients were compared with HIV infected patients co-infected with hepatotropic viruses (*p* = 0.866). | [167] |
|  | Barcelona | 2013 | Blood donors | IgM  ^2^10/216 (4.6%)  ^4^ 4/216 (1.8%)  IgG  ^2^ 116/1,082 (10.7%)  ^4^ 216/1,082 (19.9%) | IgM/IgG anti-HEV  (ELISA^2,4^) | HEV IgG prevalence showed different results depending on the IgG assay used. Significantly higher positivity rates were observed in male donors versus female donors (p < 0.05). IgG seroprevalence increased proportionally to the donors’ age (p < 0.001). | [47] |
|  | Madrid | 2014 | HIV infected patients (a), HCV infected patients (b), and HIV/HCV infected patients (c), healthy population (d) | IgM  a – 1/25 (4%)  b - 4/47 (8.5%)  c – 2/10 (20%)  d – 4/24 (16.67%)  IgG  a - 25/200 (12,5%)  b- 47/200 (23,5%)  c - 10/25 (40%)  d - 24/200 (12%) | IgG/IgM anti-HEV  (ELISA^5^) | The higher seroprevalence was found in HIV-HCV co-infected patients.  HIV infected patients have no increased risk for HEV infection. | [76] |
|  | Madrid | NA | Immunocompromised patients with inflammatory bowel disease | IgM 2/75 (2.7%)  IgG 1/80 (1.3%) | IgM/IgG anti-HEV (ELISA^12^, Immunoblot^10^ ) | A total HEV seroprevalence of 1.14% was found in patients with inflammatory bowel disease, that is similar to general population. Authors concluded these patients have no higher rate of HEV infection than the general population. | [157] |
|  | Leon | 2008-2014 | Patients with acute hepatitis E | IgM  22/23 (95.65%)  IgG  23/23 (100%) | IgM/IgG anti-HEV (ELISA^12^ immunoblot^10^) | The most of cases (N = 22) were autochthonous but one was associated with travel (China). Autoimmune markers were positive in 43.5% of the patients (complicates the diagnosis). Two subjects were classified as previous chronic liver disease and acute on-chronic liver failure, one died and the other underwent liver transplantation. | [77] |
|  | Cordoba | 2013 - 2014 | HIV infected patients and seronegative for HEV | IgM 5/627 (0.8%)  IgG 36/627 (5.7%) | IgM/IgG anti-HEV  (ELISA^4^) | Longitudinal study (median 11.96 months) that aimed the evaluation of HEV seroconversion. The incidence rate was 7.2 per 100 patients/year. Live in rural habitat was strongly associated with HEV seroconversion. | [78] |
|  | Málaga | 2016 | Patient with fulminant hepatic failure due to HEV | IgM positive  IgG positive | IgM/IgG anti-HEV  (ELISA^NA^) | Case of acute liver failure in a liver transplant patient who required a liver retransplant 9 years after receiving the original transplant. | [79] |
|  | Galicia | NA | Patient with acute hepatitis and neurological symptoms | IgM positive | IgM anti-HEV (ELISA ^12^) | First report in Spain of a simultaneous acute meningitis and hepatitis, secondary to autochthonous HEV infection in an immunocompetent. IgM anti-HEV was detected in serum and HEV RNA in serum and cerebrospinal fluid. Authors suggest HEV infection as a cause of meningitis. | [50] |
|  | Madrid | 2010-2017 | Three lung transplant recipients with symptoms and alterations in liver enzymes (a,b,c) | IgM  a – positive  b – positive  c – negative  IgG  a – positive  b – positive  c – negative | IgM/IgG anti-HEV (ELISA) | Patients with no history of travel outside Spain. HEV infection was confirmed in all patients by presence of RNA HEV in sera. One patient (not treated/ribavirin) died of progressive hepatic disease (post-mortem diagnosis of HEV infection complications). | [158] |
|  | Cordoba | 2012-2014 | HIV infected patients according to PROGINS: homozygous wild-type (a), heterozygous (b), homozygous (c) | Total (IgM/IgG)  a – 198/311 (63.7%)  b – 91/311(29.3%)  c – 22/311 (7.1%) | IgM/ IgG anti-HEV  (ELISA^4^, immunoblot^3^) | Homozygous PROGINS genotype was associated with a lower HEV seroprevalence. This effect was greater in women. None of PROGINS homozygous with recent HEV infection (IgM and/or HEV RNA) presented symptoms. Authors concluded that PROGINS mutation plays a protective role against HEV infection. | [42] |
|  | Nationwide | 2012-2014 | HIV/HCV infected patients (a), HIV infected patients (b), healthy population (c) | IgM  a – 10/198 (5.1%)  b – 3/36 (8.3%)  c – 0/30 (0%)  IgG  a - 38/198 (19.2%)  b - 4/36 (11.1%)  c - 2/30 (6.7%) | IgM/IgG anti-HEV  (ELISA^13^, immunoblot^3^) | HIV/HCV-coinfected patients have a high prevalence for IgG anti-HEV, resolved hepatitis E, and exposure to HEV; particularly patients with CD4+ <350 cells/mm3. No significant association between HEV seroprevalence and biomarkers of liver disease was found. | [169] |
|  | Ciudad Real | 2017-2018 | Four blood donors with HEV infection | Baseline  IgM 0/4  IgG 0/4  Follow up (2-4 mo later)  IgM 1/4  IgG 4/4 | IgM/IgG anti-HEV  (ELISA^4^) | Anti-HEV was not detected in any of the donors at the baseline, which lends support to the use of NAT for HEV RNA screening of blood donations, rather than determination of anti-HEV. | [48] |
|  | Zaragoza | 2011-2018 | Patients with clinical or epidemiological suspicion of hepatitis E | IgM 117/1166 (10%) | IgM anti-HEV  (ELISA^12^) | Out of 117 IgM positive patients, two (immunocompetent) presented acute non-traumatic neurologic injury in brachial plexus compatible with Parsonage-Turner syndrome and were the main focus of this study. Both registered high IgM and IgG anti-HEV indexes, and low IgG avidity, compatible with recent acute hepatitis E. An autochthonous acute hepatitis E was confirmed by the presence of RNA HEV. | [18] |
|  | Barcelona | 2014-2018 | Patient with viral acute hepatitis | IgM and/or RNA  18/100 (18%) | IgM anti-HEV (ELISA ^2^) | HEV was the second most common causes of acute hepatitis. Four of the 18 (22%) acute HEV infections occurred in immigrants. No relationship was found between immigrant status or sexual risk behaviour and acute HEV infection (IgM and/or RNA). | [80] |
|  | Barcelona | 2014-2018 | Patients with chronic autoimmune hepatitis (a), acute autoimmune hepatitis (b), acute non-autoimmune hepatitis (c) | IgM  a – 1/50 (2%)  b - 0/30 (0%)  c – 4/25 (16%)  IgG  a - 5/50 (10%)  b - 5/30 (17%)  c - 1/25 (4%) | IgM/IgG anti-HEV  (ELISA ^2^) | Seroprevalence of anti-HEV IgG was higher in patients with acute autoimmune hepatitis. These patients were older (*p* = 0.006), had higher IgG levels (*p* = 0.03) and antismooth muscle antibodies titres (*p* = .045), and were more likely to have another autoimmune disease (*p* = 0.03). The high HEV seroprevalence, high levels of gammaglobulins and high antibody titres in these patients lead authors to suggest the presence of cross-reactivity between HEV and liver antigens. | [150] |
|  | Catalonia | 2014-2019 | Patients with self-limited acute hepatitis E (a), HEV RNA-positive blood donors(b) | Baseline:  IgM  a – ^(2)^25/25 (100%)  b – ^(2,4)^12/50 (25%)  IgG  b – ^(2)^14/50 (29%)  HEV Antigen  b – ^(4)^18/50 (36%)  End of follow up (34 months):  IgM  a – ^(2)^14/25 (56%)  a – ^(4)^6/25 (24%)  IgG  a – ^(2)^21/25 (84%)  HEV Antigen  a – 0/25 (0%) | IgM/IgG anti-HEV, HEV antigen (ELISA^2,4^) | First study reporting the long-term durability of anti-HEV IgM after a self-limited acute hepatitis E. Persistence of IgM differed according to the assay being >50% (Mikrogen), opposed to 0% for HEV Antigen. HEV Antigen had the best performance (positive predictive value of 100% and diagnostic accuracy of 57%). and could be useful in settings where HEV RNA is not available. | [43] |
|  | Nationwide | 2016-2018 | Patients with drug-induced liver injury (DILI) (a), health population (b) | IgM  a – 12/144 (8.3%)  IgG  Total 131/373(35%)  a – 89/265 (34%)  b – 42/108 (39%)  HEV Antigen  a – 3/144 (2.08%) | IgM/IgG anti-HEV  (ELISA^4^) | Similar anti-HEV IgG seroprevalence rates were found in patients with suspected DILI and health population (34% and 39% respectively). Three cases were considered as having a recent hepatitis E (IgM anti-HEV and antigen positive). Authors suggests a search for active HEV infection in patients with suspected DILI. | [54] |
|  | Barcelona | 2017-2018 | MSM (a) and non-MSM (b) with acute hepatitis A | IgM  a – 1/59 (2%)  b – 1/26 (3.8%)  IgG  a – 4/59 (7%)  b – 2/26 (8%) | IgM/IgG anti-HEV  (ELISA^2^) | This study suggests that MSM are not at a higher risk for HEV infection. The low HEV seroprevalence lead authors to concluded that HEV does not spread by sexual contact or person‐to‐person. | [163] |
|  | Catalonia | 2017-2020 | HEV RNA positive blood donors | At the time of donation (baseline):  IgM 63/151 (42%)  IgG 45/151 (30%) | IgM/IgG anti-HEV (ELISA^2^) | Long-term follow-up (6 months to >1 year) was performed. At 6 month 79% (30/38) were still IgM positive and 100% remained IgG positive. | [17] |
|  | Cordoba | 2012-2020 | HIV/HCV-coinfected cirrhotic patients | Baseline:  IgM 0/75 (0%)  IgG 13/75 (17.3%)  Follow up  IgM 2/75 (2.7%)  IgG 10/75 (13.3%) | IgM/IgG anti-HEV (ELISA^2^) | Follow-up of 5.1 years was performed.  HEV seropositivity was found common in HIV/HCV-coinfected cirrhotic patients. IgG seroreversions and IgM intermittence were detected, leading authors to concluded that the use of antibodies for the diagnosis of HEV infection in this population is limited. | [81] |

MSM – Men who have sex with man; ND – Not determined; NA – Not available; HIV – Human immunodeficiency virus; HBV – Hepatitis B virus; HCV – Hepatitis C virus; PROGINS - mutations in the progesterone receptor; NAT – nucleic acid test; AST – aspartate aminotransferase

1 Abbott, North Chicago, IL, USA; 2 recomWell® HEV IgM/recomWell® HEV IgG (Mikrogen, Diagnostik, Germany); 3 recomLine® HEV IgG/IgM (Mikrogen, Diagnostik, Germany); 4Wantai™ HEV-IgM/IgG (ELISA) kit (Wantai Biological, China); 5 HEV (ELISA) MPD® HEV (ELISA) (MP Diagnostics™, MP Biomedicals, USA); 6 EUROIMMUN Medizinische Labordiagnostika AG, Lübeck, Germany; 7 Genelabs Incorporated, Redwood City, California, USA; 8 bio(ELISA)® HEV IgG/IgM (Biokit™, Spain); 9 Immunoblot GLD HEV (Genelabs Diagnostics®, Singapore); 10 recomBlot HEV IgG/IgM, Mikrogen GMBH, Martinsried, Germany; 12 HEV IgM/IgG DIA.PRO, Diagnostic Bioprobes, Milan, Italy; 13 Abbia HEV IgM/IgG AB Diagnostic Systems GmbH, Germany; 14 In house (ELISA)

15 HEV IgM/IgG Fortress Diagnostics Limited, UK; 16 Radim SpA, Roma, Italy

**Supplementary Table 3.** HEV molecular studies in animal samples, Iberian Peninsula.

| **Country** | **Area of study** | **Sampling date** | **Population details** | **Type of sample** | **Number HEV positive samples /total tested (%)** | **HEV RNA detection method** | **HEV genome target region** | **HEV genotype/ subgenotype** | **Additional data** | **References** |
| --- | --- | --- | --- | --- | --- | --- | --- | --- | --- | --- |
| Portugal | Centre (Aveiro, Coimbra, Guarda, Leiria, Viseu) | 2010-2011 | Pigs: Weaners (a), growers (b), fatteners (c), sows (d) | Stools | a - 16/50 (32%),  b - 10/50 (20%),  c - 16/50 (32%),  d - 2/50 (4%) | RT-qPCR | ORF 2 | 3 | HEV3 is highly circulating in Portuguese pig farms and in all the four age groups. Slaughter ages (weaners and fatteners) were the group with the highest HEV shedding | [83] |
|  | Centre (Aveiro, Coimbra, Guarda, Leiria, Viseu) | 2010-2011 | Pigs: Weaners (a), growers (b), fatteners (c), sows (d) | Stools | a- 12/40 (30%),  b - 8/40 (20%),  c- 12/40 (30%),  d - 2/40 (4%) | RT-qPCR | ORF 3 | ND | Estimated transmission rate parameters from five farms in Portugal: transmission rate (0.037 day^-1^), average infectious period (101 days), reproductive number R_0_ (3.7) | [84] |
|  | North (Bragança, Guarda) and Centre (Viseu, Vila Real) | 2011-2012 | Wild boars | Liver (a), stools (b) | a- 20/80 (25%),  b - 4/40 (10%) | Nested RT-PCR | ORF 1 | 3e | First report of HEV3 in sylvatic and captive wild boar destined for human consumption in Portugal | [85] |
|  | Alentejo (Portalegre, Santarém) | 2013 | Wild boars | Bile (a), stools (b) | a – 0/29 (0%)  b – 0/29 (0%) | RT-qPCR | ORF 2 | ND | None of the wild animals had an active HEV infection | [109] |
|  | Alentejo, Center, Lisbon and Tejo Valley | 2018-2020 | Deer:  red deer (a)  fallow deer (b) | Stools | a – 2/95 (2.1%)  b – 0/35 (0%) | RT-qPCR | ORF 1 | 3e | The first report of HEV in free living red deer in Portugal. | [86] |
|  | North, South, Centre | 2018-2022 | Wild boars | Stools | 4/144 (2.8%) | RT-PCR | ORF 1 | 3e, 3m | The first report of HEV subgenotype 3m in wild boar in Portugal. Study found no association between zoonotic diarrheagenic protist and HEV. | [23] |
| Spain | Barcelona | NA | Pigs | Sera (a), stools (pools) (b) | a – 0/48 (0%),  b – 0/6 (0%) | Nested RT-PCR | ORF 2 | ND | No RNA HEV was found despite pigs belonged to herds with high levels of HEV seroprevalence | [32] |
|  | Barcelona | NA | Pigs | Sera (a), stools (pools) (b) | a - 0/73 (0%),  b - 6/12 (50%) | Nested RT-PCR | ORF 2 | 3 | One new swine HEV strain, closely related to genotype 3, was identified | [87] |
|  | Valencia | 2002-2004 | Pigs: Suckling piglets (a), weaners (b), first month of feeding (c), second month of feeding (d), third month of feeding (e), breeding sows (f); boars (g) | Stools | Total: 34/146 (23.29%)  a - 2 /18 (11.1%),  b- 10/24 (41.7%),  c - 12/20 (60.0%),  d - 1/20 (5.0%),  e - 2/28 (7.1%),  f - 7/32 (21.9%),  g - 0/4 (0%) | Nested RT-PCR | ORF 2 | ND | The production stages in which most pigs excreted HEV were weaners and pigs in the first month of feeding. | [88] |
|  | Valencia | 2002-2004 | Pigs: 0-4 weeks (a), 5-12 weeks (b), 13-20 weeks (c), 21-24 weeks (d), >25 boars (e), >25 sows (f) | Sera, stools | Sera:  Total 18/131 (14%)  a – 4/20 (20%)  b – 7/22 (32%)  c – 2/20 (10%)  d – 3/27 (11%)  e – 0/4 (0%)  f – 2/38 (5%)  Stools:  Total 21/131 (16%)  a – 2/20 (10%)  b – 9/22 (41%)  c – 1/20 (5%)  d – 2/27 (7%)  e – 0/4 (0%)  f – 7/38 (18%) | Nested RT-PCR | ORF 2 | 3 | HEV was detected at a high rate in sera and stools of young pigs and also in stools of breeding sows | [172] |
|  | Barcelona | 2003-2004 | Pigs (1-4 months) with different pathological conditions | Sera (a), liver (b), bile (c), mesenteric lymph node (d), stools (e) | Total 26/69 (37.7%)  a - 7/69 (10.1%),  b- 10/69 (14.5%),  c - 13/68 (19.1%),  d - 12/69 (17.4%),  e - 8/65 (12.3%) | Nested RT-PCR | ORF 2 | 3 | A total of 22 of 69 (31.9%) pigs had mild to moderate hepatitis and 15 of them were HEV RT-PCR positive in at least one of the tested samples. Liver, mesenteric lymph node and bile presented the highest sensitivity of viral detection (samples that cannot be obtained from live pigs) | [89] |
|  | Barcelona | NA | Pigs | Sera from pigs with postweaning multisystemic wasting syndrome (PMWS) and hepatitis (a), pigs with PMWS and without hepatitis (b), pigs without PMWS and with hepatitis c) and pigs without PMWS and without hepatitis (d) | a - 21/62 (33.9%),  b - 4/48 (8.3%),  c - 9/13 (69.2%),  d - 10/33 (30.3%) | Semi-nested  RT-PCR | ORF 2 | 3 | This study indicates that swine HEV infection can be a significant contributor to the development of moderate hepatitis in pigs regardless of the PMWS status. | [111] |
|  | NA | 1998-2000 | Pigs: 3-6 weeks (a), 8-10 weeks (b), 12-13 weeks (c), 22 weeks (d), sows (e) | Sera, stools | Sera:  a - 0/19 (0%),  b - 4/6 (66.7%),  c - 13/15 (86.7%),  d - 0/10 (0%),  e - 1/16 (6.3%);  Stools:  a - 0/8 (0%),  b - 5/5 (100%),  c - 2/10 (40%),  d - 0/5 (0%),  e - 0/13 (0%) | Semi-nested  RT-PCR | ND | 3 | Viraemia was mostly detected in animals aged 8–13 weeks and HEV faecal shedding was detected in pigs of the same age | [112] |
|  | South-Central (Albacete, Cuenca, Guadalajara, Guadiana, Montes de Toledo, Ruidera, Sierra Morena, Toledo) | 2000-2005 | Wild boars: juvenile (a), sub-adult (b), adult (c) | Sera | No fencing/ no management  (total 10/51, 19.6%):  a – 2/16 (12.5%)  b – 5/15 (33.3%)  c – 3/20 (15%)  Fencing/ artificial feeding  (total 10/56, 17.9%):  a – 7/17 (41.2%)  b – 2/19 (10.5%)  c – 1/20 (5%)  Livestock-like management  (total: 7/31, 22.6%):  a – 1/5 (20%)  b – 3/11 (27.3%)  c – 3/15 (20%) | Semi-nested  RT-PCR | ORF 2 | 3 | First report of HEV3 infection in wild boar in Spain. HEV prevalence in geographical areas ranged from 7.7% (Toledo) to 40% (Guadalajara), but no statistical difference was observed. Similarly, no difference was observed concerning management system. HEV prevalence was higher in juveniles (26.3%) than sub-adult (22.2%) and adult (12.7%), and higher in females (16.2%) than in males (14.1%) but no statistical differences were observed. | [90] |
|  | Northeast | NA | Sows (a), piglets: 3 weeks (b), 6 weeks (c), 9 weeks (d), 12 weeks (e), 15 weeks (f), 18 weeks (g) | Sera, bile, liver, mesenteric lymph nodes, stools | Sera  a – 2/13 (15.4%)  Total piglets: 8/28 (28.6%)  b – 1/3 (33.3%)  c – 1/5 (20%)  d – 0/5 (0%)  e – 3/5 (60%)  f – 3/5 (60%)  g – 0/5 (0%)  Bile  Total piglets: 10/28 (35.7%),  b - 0/3 (0%)  c - 0/5 (0%)  d - 0/5 (0%)  e - 5/5 (100%)  f - 5/5 (100%)  g - 0/5 (0%)  Liver  Total piglets: 10/28 (35.7%)  b - 0/3 (0%)  c - 0/5 (0%)  d - 0/5 (0%)  e - 4/5 (80%)  f - 4/5 (80%)  g - 2/5 (40%)  Mesenteric lymph  Total piglets: 9/28 (32.1%)  b - 0/3 (0%)  c - 0/5 (0%)  d - 1/5 (20%)  e - 4/5 (80%)  f - 4/5 (80%)  g - 0/5 (0%)  Stools  Total piglets: 13/28 (46.4%)  b - 0/3 (0%)  c - 0/5 (0%)  d - 1/5 (20%)  e - 5/5 (100%)  f - 5/5  g - 2/5 | Semi-nested  RT-PCR | ORF 2 | 3 | HEV RNA was detected in serum at all ages (highest prevalence at 15 weeks of age), in stools and lymph nodes at 9 weeks of age (peak at 12 and 15 weeks). The same pig can be infected with at least two different strains of HEV during its productive life | [113] |
|  | Catalonia | NA | Chickens | Sera from flocks (a), sera bank (b) | a – 2/262 (0.76%)  b - 5/300 (1.6%) | Nested RT-PCR | ORF 2 | ND | The first description of avian HEV from chickens in Spain. Related to avian HEV strains of North America but from a separate distinct cluster | [97] |
|  | North, Centre and South | 2000–2009 | Iberian red deer | Sera | 11/81 (13.6%) | RT- PCR | ORF 2 | 3 | HEV prevalence ranged from 4.5% - 38.5% and did not differ by geographic area and management conditions (open, fenced and farmed). | [125] |
|  | Catalonia | NA | Pigs: pre-farrowing (a), breeding (b), slaughterhouse (c) | Sera, stools, liver, bile | Sera:  a - 0/12 (0%)  Stools:  a- 2/12 (17%),  b - 5/31 (16%)  Liver:  c - 6/96 (6%)  Bile  c - 5/80 (6%) | RT-PCR | ORF2 | 3 | First longitudinal survey on swine HEV infection dynamics conducted in different farrow-to-finish farms | [116] |
|  | Aragon (Huesca, Saragossa, Teruel), Catalonia (Lerida), Castilla y Leon (Salamanca), Andalusia (Jaen) | NA | Pigs: adult (a), young (b) | Sera | Total: 64/341 (18.8%)  a – 28/201 (13.9%)  b – 36/140 (25.7%) | Nested-RT-PCR | ORF 2, 3 | 3 | More positive pigs on farrow-to-finish than on grower/fattener farms (60% vs. 38.1%), but without significant difference (*p* = 0.066) | [117] |
|  | NA | 2010 | Pigs | Stools (a), liver (b), meat (c), sausage (d) | a - 15/39 (38%),  b - 1/39 (3%),  c - 0/39 (0%),  d - 6/93 (6.4%) | RT-qPCR | ORF 2 | 3f | The presence of HEV in sausages sampled at processing and point of sale demands containment measures | [98] |
|  | ND | 2010-2011 | Pigs: sows (a), boars (b) | Stools | a – 0/144 (0%)  b – 1/23 (4.3%) | RT-PCR | ORF 3 | NA | Transmission dynamics of HEV not determined because almost all animals were HEV negative | [84] |
|  | Barcelona | 2012 | Pigs | Piece of meat | NAp | RT-qPCR | ORF 2 | 3f | Ingestion of the cooked pork meat responsible for severe acute hepatitis E in an immunocompromised patient (confirmed by phylogenetic analysis) | [91] |
|  | Castilla-La Mancha | 2003-2010 | Pigs (a), wild boars (b), red deer (c) | Sera | a - 0/48 (0%),  b - 16/158 (10.1%),  c - 13/81 (16.1%) | Nested RT-PCR | ORF 3 | 3 | A constant presence of HEV in wild boar and deer was observed among all study years (2003–2010) | [120] |
|  | Andalusia | 2015 | Wild boars | Piece of meat | NAp | RT-qPCR | ORF 2 | 3 | Wild boar meat consumption responsible for familial HEV outbreak (confirmed by phylogenetic analysis) | [51] |
|  | Andalusia | 2015- 2016 | Wild boars | Liver (a), sera (b) | a - 4/58 (6.8%),  b - 1/58 (1.7%) | RT-qPCR | ORF 2, 3 | ND | This study shows in nonviremic animals evidence of HEV liver infection without hepatitis lesions. The possibility of consumption of contaminated liver of HEV seronegative animals is suggested | [92] |
|  | Andalusia (Cadiz) | 2015-2016 | Black rats | Liver | 2/50 (4%) | RT-qPCR | ORF 1, 2, 3 | *Orthohepevirus* *C-*1 | First detection of rat HEV in Black rats from Spain | [99] |
|  | Cordoba | 2015 | Pigs (fatteners and sows) | Sera | 172/1040 (16.5%) | RT-qPCR | ORF 2, 3 | ND | Factors associated with higher prevalence of HEV infection were: extensive farming (23.9%), absence of sanitary ford (33.8%), no quarantine period (20.8%) and contact with domestic species (24.5%) | [93] |
|  | Andalusia | 2015-2016 | Wild boars (hunting season October 15^th^ – February 15^th^) | Sera | 33/142 (23.2%) | RT-PCR | ND | ND | Prevalence may depend on the season when the animal was hunted, and the potential risk of zoonotic transmission may therefore fluctuate | [94] |
|  | Andalusia | 2010-2014 | Equines: horses (a), donkeys (b), mules (c) | Sera | a - 3/692 (0.4%),  b - 1/86, (1.2%),  c - 3/83 (3.6%) | RT-qPCR | ORF 2 | 3 e, f, g | First survey of HEV in equids in Europe and to report HEV infection in donkeys and mules worldwide. HEV was more prevalent in mules compared with horses and was higher in geriatric animals than in adults and young animals. | [100] |
|  | Andalusia (Doñana National Park) | 2015 | Wild boars | Sera | 1/99 (1%) | Nested RT-PCR | ORF 2 | 3r | The strain detected belonged to the propose HEV subtype 3r and it can be considered an emerging zoonotic subtype in Europe | [95] |
|  | Barcelona | 2015-2016 | Wild boars | Sera (a), stools (b) | a - 31/190 (16%),  b - 25/207 (12%) | Nested RT-PCR | ORF 1 | 3 f, c, i | One strain was genetically similar to HEV found in human | [121] |
|  | Nationwide | 2017 | Pigs | Sera (a), stools (b), heart (c), kidney (d), liver (e), ribs (f), bacon (g), diaphragm (h), lean ham (i), loin head (j) | a – 3/45 (6.7%),  b– 6/45 (13.3%),  c– 4/45 (8.9%),  d – 5/45 (11.1%),  e – 7/45 (15.6%),  f – 0/45 (0%),  g – 0/45 (0%),  h – 1/45 (2.2%),  i – 0/45 (0%),  j- 0/45 (0%) | RT-qPCR | ORF 2 | 3f | This is the first report on detection of HEV RNA in kidney and heart samples (which shows extrahepatic dissemination) of naturally infected and apparently healthy pigs | [122] |
|  | Andalusia | 2013-2016 (rabbits) 2018 (hares) | Wild rabbits (a), Iberian hares (b) | Liver | a - 0/372 (0%),  b - 0/78 (0%) | RT-qPCR | ORF 2 | ND | Absence of HEV circulation in European wild rabbit and Iberian hare populations during the study period and suggest a limited risk of transmission of HEV from these wild lagomorph species to other mammals, including humans | [101] |
|  | Andalusia | 2015 | Pigs | Sera (a), stools from pen floor (b) | a- 177/1040 (17%)  b - 383/1040 (36.8%) | RT-qPCR | ORF 3 | ND | The effectiveness of a non- invasive screening approach (stools from pen floor) was used to determining the HEV status of 26 pig farms under different management systems. HEV RNA was positive in 70.6% (12/17) intensive farms and 100% of extensive farms (9/9) | [171] |
|  | Cordoba | 2015-2016 | Black Iberian pigs (a), wild boars (b) | Sera, stools | Sera:  a – 31/186 (16.7%)  b – 24/142 (16.9%)  Stools:  a – 19/186 (10.2%)  b – 11/142 (7.7%) | RT-qPCR | ORF2 | ND | High prevalence of enteroparasites in black Iberian pigs and wild boars was found. Animals carrying *Giardia duodenalis* and *Blastocystis* sp. infections showed a significantly lower rate of HEV infection than those not harbouring these enteroparasites (P < 0.001). Authors suggested that extracellular *Giardia duodenalis* and *Blastocystis* sp. might have a protective effect on HEV acquisition in swine | [102] |
|  | Andalusia, Aragón, Castilla y León, Castilla-La Mancha, Catalonia, Extremadura, Galicia, Murcia | NA | Pigs (a), Iberian pigs (b), wild boars (c) | Sera, liver transudate | Sera:  Total: 13/58 (22.4%)  a- 8/35 (22.8%),  b – 0/5 (0%),  c – 5/18 (27.7%)  Liver transudate:  Total: 4/58 (6.9%)  a – 0/35 (0%)  b – 0/5 (0%),  c – 4/18 (22.2%) | RT-qPCR | ORF 3 | ND | Liver transudate could be used for HEV RNA detection | [104] |
|  | Córdoba | 2015 | Wild boars (a),  ticks (b) | Sera (boar), whole tick | a – 10/29 (34.5%)  b – 11/29 (37.9%) | RT-qPCR,  RT-PCR | ORF 3  ORF 2 | 3f | First detection of HEV RNA in *Hyalomma lusitanicum* ticks feeding on wild boars | [105] |
|  | Córdoba | 2017-2020 | Dogs (a), cats (b) | Sera | a – 0/152 (0%  b – 0/144 (0%) | RT-qPCR | ORF 1 | ND | The first study investigating HEV RNA of *Orthohepevirus* species (HEV-A, HEV-HEV-C) in dogs and cats. RNA was not detected, despite seropositivity of same animals. | [106] |
|  | Córdoba | 2015 | HEV co-infected pigs: large white (a), Iberian pigs intensive (b), Iberian pigs extensive (c) | Stools | HEV Positive:  *Balantioides coli*  Total: 11.1%  a – 12.8%  b – 1.8%  c – 16.7%  *Blastocystis* spp  Total: 13.7%  a – 12.7%  b – 3.1%  c – 19.5%  *Cryptosporidium* spp  Total: 30.8%  a – 40%  b – 0%  c – 66.7%  *Enterocytozoon bieneusi*  Total: 24.2%  a – 30.4%  b – 0%  c – 100%  *Giardia duodenalis*  Total: 7.8%  a – 10.7%  b – 8.3%  c – 0%  HEV Negative  *Balantioides coli*  Total: 14.7%  a – 17.8%  b – 8.3%  c – 17.6%  *Blastocystis* spp  Total: 12.5%  a – 19.7%  b – 6.5%  c – 14.9%  *Cryptosporidium* spp  Total: 12%  a – 13%  b – 6.1%  c – 16.2%  *Enterocytozoon bieneusi*  Total: 12.2%  a – 13%  b – 6.1%  c – 16.7%  *Giardia duodenalis*  Total: 13.7%  a – 16%  b – 5.5%  c – 18.6% | RT-qPCR | ORF 3 | 3f | Study provides a scientific ground for a novel modulatory effect involving intracellular enteroparasites. Study shows that the presence of the extracellular enteroparasites show a protective effect on the risk of HEV acquisition, whereas intracellular enteroparasites seem to have the opposite effect, favouring the HEV infection. | [103] |
|  | Central, South, Southwest | 2010-2021 | Iberian lynx (*Lynx pardinus*) | Serum (a), liver (b), stools (c), | a – 0/248 (0%)  b – 0/158(0%)  c – 1/73 (1.4%) | RT-qPCR, nested RT-PCR | ORF1  ORF2 | 3f | First molecular report of HEV-A infection in free-ranging felines. HEV RNA was detected in stools of one (1/364; 0.3%) free-ranging adult animal. | [107] |
|  | Andalusia | 2015-2017 | Sheep (a), goats(b) | Sera | a - 0/240 (0%)  b - 0/240 (0%) | RT-PCR | ORF 2 | Nap | First study to assess the presence of HEV RNA in small ruminants in Spain | [108] |
|  | Andalusia | 2018-2021 | Wild boar | Sera (a), liver (b), hepatic lymph nodes (c), testis (d) | a – 4/191 (2.09%)  b – 3/4  c – 3/4  d – 1/4 | RT-qPCR  Nested RT-PCR | ORF 2  ORF 1 | 3f | Study aims to evaluate the presence of HEV 3 in testis of naturally infected wild boars. HEV 3 was detected in testis but no tissue damage was observed. It was not possible to discard semen as a potential source of HEV transmission in suids. | [96] |

NA - not available; Nap – Not applicable; ND – Not determined

**Supplementary Table 4.** HEV serological studies in animal samples, Iberian Peninsula.

| **Country** | **Area of study** | **Sampling date** | **Population details** | **Number anti -HEV positive samples /total tested (%)** | **HEV serological assay** | **Additional data** | **References** |
| --- | --- | --- | --- | --- | --- | --- | --- |
| Portugal | Alentejo (Portalegre, Santarém) | 2013 | Wild boars | IgM/IgG/IgA  4/29 (14%) | IgM/IgG/IgA anti-HEV^2^ | First serological report evidencing HEV infection in wild boars from Portugal | [109] |
|  | Alentejo (Beja) | 2014-2015 | Wild rabbits | IgM/IgG  3/74 (4.1%) | IgM/IgG anti-HEV^3^ | First detection of anti-HEV antibodies in Portuguese wild rabbits | [110] |
|  | Centre | 2016 | Sheep | IgG  15/90 (16.6%) | IgG anti-HEV Ab^5^ | First detection of anti-HEV antibodies in Portuguese sheep. | [64] |
| Spain | Catalonia | NA | Sows (a), boars (b) and piglets (c) | IgG  a– 6/26 (23.1%)  b - 7/24 (29.1%)  c – 2/10 (20%) | IgG anti-HEV^1^ | The global HEV seroprevalence of pigs from these commercial herds was 25% | [32] |
|  | Catalonia | NA | Pigs | IgG  10/73 (13.7%) | IgG anti-HEV^4^ | Seropositive animals were all >10 weeks (fattening pigs and sows) | [87] |
|  | Barcelona | NA | Sera from pigs with postweaning multisystemic wasting syndrome (PMWS) and hepatitis (a), pigs with PMWS and without hepatitis (b), pigs without PMWS and with hepatitis c) and pigs without PMWS and without hepatitis (d) | IgG  a - 37/64 (57.8%),  b- 20/50 (40.0%),  c - 3/13 (23.1%),  d - 10/33 (30.3%) | IgG anti-HEV^1^ | Seropositivity to HEV was more likely to occur in pigs with hepatitis compared to pigs without hepatitis | [111] |
|  | NA | 1998-2000 | Pigs: 3-6 weeks (a), 8-10 weeks (b), 12-13 weeks (c), 22 weeks (d), gilts/sows (e) | Total IgM 118/418 (28.2%)  a - 13/75 (17.3%), b - 17/72 (19.4%), c - 40/123 (28.0%), d - 27/54 (50.0%),  e - 24/74 (32.4%);  Total IgG 184/439 (41.9%)  a- 40/85 (47.1%), b - 34/95 (35.8%), c - 45/126 (35.7%), d - 20/59 (33.9%),  e - 45/74 (60.8%) | IgM/IgG anti-HEV^1^ | The highest proportion of IgG positive pigs comprised the adult sows (60.8%), followed by young piglets aged 3–6 weeks (47.1%). IgM positive pigs were more frequently found in animals ≥12 weeks of age | [112] |
|  | South-Central (Albacete, Cuenca, Guadalajara, Guadiana, Montes de Toledo, Ruidera, Sierra Morena, Toledo) | 2000-2005 | Wild boars: juvenile (a), sub-adult (b), adult (c) | Total IgM/ IgG/IgA 64/150 (42.7%)  IgM 32/150 (21.3%)  IgG 42/150 (28%)  IgA 34/150 (22.6%)  No fencing/ no management (IgM/IgG/IgA)  (Total 20/51, 39.2%):  a – 5/16 (31.2%)  b – 7/15 (46.7%)  c – 8/20 (40%)  Fencing/ artificial feeding (IgM/IgG/IgA)  (Total 22/57 38.6%):  a – 6/17 (35.3%)  b – 7/20 (35%)  c – 9/20 (45%)  Livestock-like management (IgM/IgG/IgA)  (Total: 22/42, 52.4%):  a – 5/5 (100)  b – 8/18 (44.4%)  c – 9/19 47.4%) | IgM/IgG/IgA anti-HEV^1^ | First report of HEV infection in wild boar in Spain. HEV seroprevalence in geographical areas ranged from 16.7% (Ruidera) to 80% (Guadalajara).  Wild boar under intensive management showed higher IgG and IgM seroprevalences.  Seroprevalence did not significantly differ between age classes: juveniles (42.1 %) sub-adult (41.5 %), adult (44.1%) | [90] |
|  | Northeast | NA | Sows (a), piglets: 1 weeks (b), 3 weeks (c), 6 weeks (d), 9 weeks (e) | IgM  a – 2/13 (15.4%)  IgG  a – 10/13 (76.9%)  b – 23/42 (54.7%)  c – 15/43 (32.9%)  d – 9/41 (21.9%)  e – 5/36 (5.6%)  IgA  a – 1/13 (7.7%) | IgM/IgG/IgA anti-HEV^1^ | Piglet anti-HEV IgG (maternal antibodies) was associated with sow serological status and antibody duration related to sow titres. IgG and IgA (maternal antibodies) lasted until 9 and 3weeks of age, respectively. Anti-HEV IgM and IgA seroconversion in piglets occurred by 12 weeks of age while anti-HEV IgG at 15 weeks. On individual basis, IgG was detectable until the end of the study (22 weeks) while IgM and IgA antibody duration were of 4–7 weeks. | [113] |
|  | Catalonia | 1985-1997 | Pigs | IgG  1390/2871 (48.4%) | IgG anti-HEV^1^ | HEV circulates in Spanish pig farms at least since 1985. Seroprevalence per year ranged from 25% to 71% | [114] |
|  | Catalonia | NA | Cows (a), sheep (b), goats (c), cats (d), wild rodents (e), pigs (f) | IgG  a– 0/1170 (0%)  b– 36/1357 (2.6%)  c – 18/1143 (1.6%)  d– 20/54 (37%)  e – 0/166 (0%)  f – 180/252 (71%) | IgG anti-HEV^1^ | An ELISA test based on the truncated ORF2 capsid protein from a genotype 3 strain was developed and applied for the screening of anti-HEV among various domestic animal species | [124] |
|  | Catalonia | NA | Chickens (flocks) | IgG  26/29 (89.7%) | IgG anti-HEV^1^ | Seropositive animals were detected at any age but chickens >40 weeks have higher seropositivities. | [97] |
|  | North, South, Center | 2000 – 2005  2006 – 2009 | Iberian red deer | IgG  101/968 (10.4%) | IgG anti-HEV^1^ | Seroprevalence in 2006-2009 (12.2%) was significantly higher than 2000-2005 (7.5%, *p*<0.05). Seroprevalence differed significantly by management types (*p*<0.05), with higher values in open (14.9%) than in fenced (9.1%) and farmed (2.6%) areas. Differ by geographic area from 0% to 31.4%. There was no significant difference between sex and age classes. | [125] |
|  | NA | NA | Pigs  serum (a), muscle fluid (b) | IgG  a - 43/67 (64%), b - 43/67 (64%) | IgG anti-HEV^1^ | Muscle fluid can be used as an alternative to serum for serological detection of HEV antibodies in slaughter pigs. | [115] |
|  | Catalonia | NA | Pigs  (sows) | IgM 18/119 (15%)  IgG 70/119 (59%) | IgM/IgG anti-HEV^1^ | A monitorization from nursery to slaughter was performed in some piglets. Anti-HEV IgM were firstly detected at 7 weeks of age (in 5 farms) and at 13 weeks of age in (in 1 farm). At slaughter age 50–100% of pigs had seroconverted to anti-HEV IgG (in 5 farms) but only 5% in one farm. | [116] |
|  | Aragon (Huesca, Saragossa, Teruel), Catalonia (Lerida), Castilla y Leon (Salamanca), Andalusia (Jaen) | NA | Pigs: adult (a), young (b) | IgG  Total: 233/1141 (20.4%)  a – 115/381 (30.2%)  b – 118/760 (15.5%) | IgG anti-HEV^1^ | Pigs from 85 herds (6 provinces) were study. 80% of farms had at least one IgG positive pig. Seroprevalence by province revealed a significantly unequal distribution, ranging between 2.5% and 26.4% (p < 0.001). Seroprevalence dropped between 3 and 11 weeks of age, and then rose by the 15th week. | [117] |
|  | Nationwide | 2000-2011 | Wild boars | IgG  248/942 (26.3%) | IgG anti-HEV^1^ | The overall seroprevalence remained globally stable from period 1 (2000-2005) to period 2 (2006-2011). It was affected by the type of management, being higher in fenced than open sites | [118] |
|  | Barcelona | 2009 - 2010 | Spray-dried porcine plasma | Anti-HEV  85/85 (100%) | IgG anti-HEV^1^  Total anti-HEV^1^ | A retrospective study of sera samples (from several swine trials) collected from pigs (3 to 15 weeks of age) fed diets containing 8% SDPP for 4 to 9 weeks. No HEV seroconversion was observed, suggesting that feeding SDPP in diets for pigs does not represent a risk of transmitting HEV | [119] |
|  | Toledo, Ciudad Real in Castile-La Mancha | 2003-2010 | Pigs (a), wild boars (b), red deer (c) | Anti-HEV  a – 21/48 (43.7%)  b– 62/108 (57.4%)  c – 9/70 (12.8%) | Total anti-HEV^2^ | Positive samples were distributed among all study years (2003–2010). No difference in seropositivity was observed in the different geographic regions | [120] |
|  | Andalusia | 2015 - 2016 | Wild boars: juvenile (a), sub-adults (b), adults (c) | Anti-HEV  Total: 3/58 (5.2%)  a – 1/6 (16.6%)  b – 0/15 (0%)  c – 2/37 (5.4%) | Total anti-HEV^6^ | Seroprevalence in this region (southern Spain) differs from other regions of Spain. Could be underestimated due to the low number of animals used | [92] |
|  | Catalonia, Madrid, Valencian Community, Andalusia | 2002-2018 | Non-human primates  (from zoos) | IgG  8/181 (4.4%) | IgG anti-HEV^5^ | This is the first report of seropositivity in *Varecia variegata*, *Pan troglodytes*, and *Macaca sylvanus*. Primates of the *Hominidae* family are risk factor for HEV seropositivity. Anti‐HEV antibodies were found in six of the eight zoos included in the study (75.0%) | [126] |
|  | Andalusia | 2015 | Wild boars: juvenile (a), sub-adults (b), adults (c) | Anti-HEV  Total:57/99 (57.6%)  a – 6/19 (24%)  b – 13/19 (68.4%)  c – 37/54 (68%) | Total anti-HEV^6^ | Significantly higher seropositivity was found among adults (*p*<0.001) and subadults (*p*=0.005) than in yearling animals | [95] |
|  | Barcelona | 2015-2016 | Wild boars | IgM/IgG  112/190 (59%) | IgM/IgG anti-HEV^3^ | Seroprevalence similar to others reported in central Spain | [121] |
|  | Nationwide | 2017 | Pigs | IgG  33/45 (73.3%) | IgG anti-HEV^7^ | Seropositive animals were found in all of the nine slaughterhouses evaluated in this study | [122] |
|  | Cuenca, Pontevedra, Barcelona, Saragossa, Burgos, Malaga, Cordoba, Girona, Toledo, Murcia, Badajoz, Madrid | NA | White pigs (a), Iberian pigs (b), wild boars (c) | Serum samples  Total: 85/125 (68.0%)  a - 34/44 (77.2%)  b - 29/46 (63.0%)  c - 22/35(62.8%)  Liver transudate  Total: 77/125 (61.6%)  a - 37/44 (84.0%)  b - 20/46 (43.4%)  c - 20/35(57.1%) | Total anti-HEV^7^ | Liver transudate was used as an alternative matrix to serum for the detection of anti-HEV antibodies (best results at 1:10 dilution) | [104] |
|  | Cordoba | 2005-2018 | Wild boars | IgG  327/700 (46.7%) | IgG anti-HEV^6^ | First longitudinal long-term survey (2005–2018) of HEV exposure in wild boar. Age was statistically significant to explain the seropositivity and lowest when high rainfall season | [123] |
|  | Córdoba | 2017-2020 | Dogs (a), cats (b) | Anti HEV  Total: 19/296 (6.4%)  a – 15/152 (9.9%)  b - 4/144 (2.8%) | Total anti-HEV^2^ | This study provide evidence of HEV exposure in sympatric urban cats and dogs in southern Spain | [106] |
|  | Andalusia | 2015-2017 | Sheep (a), goats(b) | Anti HEV  Total: 38/480 (7.9%)  a - 5/240 (2.1%)  b - 33/240 (13.8%) | Total anti-HEV^2^ | Significantly higher seroprevalence was found in goats compared to sheep (p>0.001). Data showed that sheep and goat are naturally but not equally exposed to HEV | [108] |
|  | Central, South, Southwest | 2010-2021 | Iberian lynx (*Lynx pardinus*) (a) free-living, (b) captive | Anti HEV  Total: 50/275 (18.2%)  a – 12/162 (7.4%)  b – 38/113 (33.6%) | Total anti-HEV^2^ | The first HEV serosurvey study in Iberian lynx. Serological results suggest widespread but not homogeneous HEV circulation in these animals.  Seroprevalence was significantly higher in captive lynx compared to free-living animals (p<0.001).  In captive Iberian lynx age (senile, adult and subadult) was identified as risk factor for HEV exposure. When these individuals were longitudinally surveyed 29.5% seroconverted against HEV. | [107] |

NA - not available; PRRSV - porcine reproductive and respiratory syndrome virus; PMWS - Post-weaning multisystemic wasting syndrome.

Serological assays: ^1^ In house, ^2^ HEV ELISA MPD® HEV ELISA (MP Diagnostics™, MP Biomedicals, USA),^3^ Axsym™, Abbott GMBH Diagnostika, Germany, ^4^ Abbott HEV EIA™, Abbott diagnostics division (USA), ^5^ Wantai™ HEV-IgG ELISA kit (Wantai Biological, China), ^6^ PrioCHECK™ ELISA Kit, porcine (Thermo Fisher Scientific, USA), ^7^ kit ID Screen Hepatitis E Indirect Multi-species™ (IDvet, France)

**Supplementary Table 5.** HEV studies in environmental samples, Iberian Peninsula.

| **Country** | **Area of study** | **Sampling date** | **Type of sample** | **Number HEV positive samples / total tested (%)** | **HEV RNA detection method** | **HEV genome target region** | **HEV genotype/ sub genotype** | **Additional data** | **References** |
| --- | --- | --- | --- | --- | --- | --- | --- | --- | --- |
| Portugal | North, Centre, Lisboa and Vale do Tejo, Alentejo, Algarve | 2013 | Influent, effluent wastewater | 2/60 (3.3%) | RT-qPCR, nested RT-PCR | ORF 2 | 3i, f | HEV detected only in influent wastewater of the treatment plants | [127] |
|  | North (Lima River estuary) | 2016-2017 | Sea urchins (gonads) | 2/4 (50%) | RT-qPCR, nested RT-PCR | ORF 2 | 3 | HEV contamination reported for the first time in sea urchins | [128] |
|  | Centre | 2019 | Surface water (a), drinking water (b) | a - 21/27 (77.8%),  b - 24/36 (66.7%) | RT-qPCR | NA | ND | HEV detected in surface and drinking water; HEV infectivity assessed | [22] |
| Spain | Barcelona | 1994-1998 | Raw sewage | 1/37 (2.7%) | Nested RT-PCR | ORF 1, 2, 3 | ND | HEV detected in sewage for the first time in Spain; HEV infectivity demonstrated by *Rhesus* monkey inoculation | [129] |
|  | Barcelona | NA | Sewage samples from porcine slaughter houses | 1/12 (8.3%) | Nested RT-PCR | ORF 2 | ND | HEV detected in pig slaughterhouse sewage highly similar to HEV detected in human sera | [32] |
|  | Valencia | NA | Coquina clams | 0/20 (0%) | Semi-nested RT-PCR | NA | ND | HEV not detected in frozen clams (imported from Peru) contaminated with human enteric viruses and associated with a large hepatitis A outbreak | [137] |
|  | Barcelona | 1994-2002 | Urban sewage | 20/46 (43.5%) | Nested RT-PCR | ORF 2 | 3 | An eight-year study of HEV in urban sewage; an increase of HEV positive samples was detected in 2001 - 2002 | [87] |
|  | Valencia | 2002-2004 | Raw manure (a), animal drink water (b) | a – 8/16 (50%), b – 1/16 (6.2%) | Nested RT-PCR | ORF 2 | ND | Samples from 16 different farms. HEV high presence in manure ditches raises concerns about its use as soil fertilizer | [88] |
|  | Barcelona | 2004-2006 | Urban sewage (a), slaughterhouse biosolids (b) river water or sludge samples from drinking-water treatment plant (c) | a– 4/4 (100%); b – 1/4 (25%),  c – 0% | Semi-nested RT-PCR | ORF 2 | 3 | HEV frequently detected in urban sewage but in low concentrations | [130] |
|  | Valencia | 2002-2004 | Manure-ditch from pigs | 10/17 (59%) | Nested RT-PCR | ORF 2 | 3 | HEV high presence in manure ditches raises concerns about its use as soil fertilizer | [172] |
|  | Barcelona | 2001-2006 | Urban sewage (a), biosolid (b), slaughterhouse (c) | a – 6/34 (17.6%), b – 2/4 (50%), c – 2/5 (40%) | Semi-nested RT-PCR | ORF 2 | 1, 3 | HEV genotype 1, similar to Indian strains, detected in urban sewage in Barcelona | [131] |
|  | Barcelona, Valencia | 2000-2008 | Sewage  Barcelona 2000–2004 (a)  Barcelona 2006–2008 (b)  Valencia 2007–2008 (c) | Total 29/91 (32%)  a - 7/18 (38.9%)  b - 9/32 (28.1%)  c - 13/41 (31.7%) | RT-qPCR, nested RT-PCR | ORF 2 | 1,3 | HEV genotype 1 was detected in Barcelona sewage, which shows the circulation (low levels) of this genotype also in industrialized countries | [72] |
|  | Galicia | 2010 | Mussel tissue | 3/51 (6%) | RT-qPCR | ORF1, 2 | ND | HEV contamination of commercial mussels at retail level | [138] |
|  | Eastern | 2006-2011 | Manure treatment type a.1:  reception pond (a.1.1), anaerobic pond (a.1.2), solid product from centrifuge (a.1.3), aerobic pond 1 (a.1.4), aerobic pond 2 (a.1.5), fermentation channel (Start) (a.1.6), fermentation channel (End) (a.1.7), maturation warehouse (a.1.8), final pellet (a.1.9);  Manure treatment type a.2:  reception pond (a.2.1), anaerobic pond (a.2.2), solid product from centrifuge (a.2.3), aerobic pond 1 (a.2.4), aerobic pond 2 (a.2.5), fermentation channel (Start) (a.2.6), fermentation channel (End) (a.2.7), maturation warehouse (a.2.8), final pellet (a.2.9);  Manure treatment type b.1:  reception pond (b.1.1), fermentation channel (Start) (b.1.2), fermentation channel (End) (b.1.3), maturation warehouse (b.1.4), final pellet (b.1.5);  Manure treatment type b.2:  reception pond (b.2.1), fermentation channel (Start) (b.2.2), fermentation channel (End) (b.2.3), maturation warehouse (b.2.4), final pellet (b.2.5);  Manure treatment type b.3:  reception pond (b.3.1), fermentation channel (Start) (b.3.2), fermentation channel (End) (b.3.3), maturation warehouse (b.3.4), final pellet (b.3.5) | a.1.1 - 24/27 (88.9%), a.1.2 - 18/27 (66.7%), a.1.3 - 3/27 (11.1%), a.1.4 - 3/27 (11.1%),  a.1.5 - 0/27 (0%), a.1.6- 0/27 (0%), a.1.7 - 0/27 (0%), a.1.8 - 0/27 (0%), a.1.9 - 0/27 (0%);  a.2.1 - 9/9 (100%), a.2.2 - 6/9 (66.7%), a.2.3 - 6/9 (66.7%), a.2.4 - 0/9 (0%), a.2.5 - 0/9 (0%), a.2.6 - 0/9 (0%), a.2.7 - 0/9 (0%), a.2.8 - 0/9 (0%),  a.2.9 - 0/3 (0%);  b.1.1 - 0/3 (0%), b.1.2 - 0/3 (0%), b.1.3 - 0/3 (0%), b.1.4 - 0/3 (0%), b.1.5 - 0/3 (0%),  b.2.1 - 3/6 (50%);  b.2.2 - 0/6 (0%), b.2.3 - 0/6 (0%), b.2.4 - 0/6 (0%),  b.2.5 - 0/6 (0%);  b.3.1 - 3/9 (33.3%), b.3.2 - 0/9 (0%), b.3.3 - 0/9 (0%), b.3.4 - 0/9 (0%), b.3.5 - 0/9 (0%) | Nested RT-PCR | ORF 2 | ND | HEV not detected in any final product (compost) destined to be commercialized as a soil fertilizer, which shows that composting is a suitable method to eliminate HEV and to reduce the transmission of HEV from pigs to humans | [139] |
|  | Barcelona | 2009 - 2010 | Spray-dried porcine plasma (SDPP) | 11/49 (22.4%) | Semi-nested RT-PCR | ORF2 | ND | First study reporting presence of anti-HEV antibodies and HEV RNA in SDPP. All the commercial SDPP analysed (85) presented anti-HEV antibodies (100%). No transmission of HEV in pigs fed with SDPP was observed | [119] |
|  | Barcelona | 2011 - 2012 | Influent raw sewage (a), secondary treatment effluent (b) | a – 5/37 (13.5%);  b – 4/32 (12.5%) | Nested RT-PCR | ORF2 | ND | HEV found at low and at similar levels in influent and effluent wastewaters | [132] |
|  | Galicia  (Ría do Burgo) | 2010-2012 | Mussel hepatopancreas | 12/81 (14.8%) | RT-qPCR | ORF 1, 3 | 3e | HEV detected in shellfish beds close to a high population density area approved for the production of shellfish for human consumption | [19] |
|  | South Catalonia | 2012 - 2013 | Lagooning influent (a), lagooning effluent (b) wastewater | a - 0/12 (0%); b – 0/12 (0%) | RT-qPCR | ORF1 | ND | HEV not detected in reclaimed water of a lagooning system during one-year period | [140] |
|  | Valencia, Murcia | 2017 | Sewage (in Valencia) (a), irrigation water samples (in Murcia) (b), lettuce (in Murcia) (c) | a - 10/14 (71.4%), b - 0/24 (0%), c - 0/36 (0%) | RT-qPCR | ORF 2 | 3f | HEV not detected in irrigation water and lettuce | [133] |
|  | Galicia  (Ría de Ares-Betanzos, Ría de Vigo) | 2011-2012 | Shellfish | 41/168 (24.4%) | RT-qPCR, nested RT-PCR | ORF 2, 3 | 3e | HEV detected at significant levels in bivalve mollusks from Galician Rías | [21] |
|  | Valencia | 2018-2019 | DWTP (two plants): influent (a.1, a.2), effluent (b.1, b.2);  WWTP (four plants): influent (c.1, c.2, c.3, c.4), effluent (d.1, d.2, d.3, d.4) | a.1 – 0/14 (0%), a.2 – 0/14 (0%),  b.1 – 0/14 (0%), b.2 – 0/14 (0%);  c.1 – 2/16 (12.5%), c.2 – 4/16 (25%), c.3 – 2/15 (13.3%), c.4 – 11/15 (73.3%), d.1 – 0/13 (0%), d.2 – 0/13 (0%), d.3 – 0/13 (0%), d.4 – 0/13 (0%) | RT-qPCR | ORF 3 | ND | HEV only detected in the influent wastewaters of the four WWTP | [134] |
|  | Barcelona | 2015 - 2016 | Drinking water (a), reservoir water (b), groundwater (c), river water (d), raw sewage (e), conventional activated sludge (f), reclaimed (wetland) (g) | a - 0/6 (0%), b - 0/6 (0%),  c - 1/12 (8%), d - 0/12 (0%),  e - 1/12 (8%), f - 0/12 (0%),  g - 0/12 (0%) | RT-qPCR | ORF3 | ND | HEV detected in groundwater where porcine contamination was identified | [135] |
|  | Barcelona | 2016 - 2017 | Distribution water (a), reservoir water (b), groundwater (c), river water (d), wetland (e), raw sewage (f) | a - 0/6 (0%), b - 0/6 (0%),  c - 1/12(8%), d - 0/12 (0%),  e - 0/12 (0%), f - 1/12 (8%) | RT-qPCR | ORF3 | ND | HEV detected in raw sewage and groundwater (in summer) using metagenomic analysis | [136] |

DWTP - drinking water treatment plant, NA – Not available, ND – Not determined, SDPP – Spray-dryer porcine plasma, WWTP - wastewater treatment plant
